# PCTAIRE1 promotes mitotic progression and resistance against antimitotic and apoptotic signals

**DOI:** 10.1242/jcs.258831

**Published:** 2022-02-09

**Authors:** Syed Qaaifah Gillani, Irfana Reshi, Nusrat Nabi, Misbah Un Nisa, Zarka Sarwar, Sameer Bhat, Thomas M. Roberts, Jonathan M. G. Higgins, Shaida Andrabi

**Affiliations:** 1 Department of Biochemistry, University of Kashmir, Srinagar 190006, India; 2 Department of Biotechnology, University of Kashmir, Srinagar 190006, India; 3 Dana Farber Cancer Institute, Harvard Medical School, Boston, MA 02115, USA; 4 Biosciences Institute, Faculty of Medical Sciences, Newcastle University, Newcastle upon Tyne NE2 4HH, UK

**Keywords:** PCTAIRE1, CDK16, PLK1, PP2A, Chemotherapeutic resistance, Mitotic arrest

## Abstract

PCTAIRE1 (also known as CDK16) is a serine-threonine kinase implicated in physiological processes like neuronal development, vesicle trafficking, spermatogenesis and cell proliferation. However, its exact role in cell division remains unclear. In this study, using a library screening approach, we identified PCTAIRE1 among several candidates that resisted mitotic arrest and mitotic cell death induced by polyomavirus small T (PolST) expression in mammalian cells. Our study showed that PCTAIRE1 is a mitotic kinase that localizes at centrosomes during G2 and at spindle poles as the cells enter mitosis, and then at the midbody during cytokinesis. We also report that PCTAIRE1 protein levels fluctuate through the cell cycle and reach their peak at mitosis, during which there is an increase in PCTAIRE1 phosphorylation as well. Interestingly, knockdown of PCTAIRE1 resulted in aberrant mitosis by interfering with spindle assembly and chromosome segregation. Further, we found that PCTAIRE1 promotes resistance of cancer cells to antimitotic drugs, and this underscores the significance of PCTAIRE1 as a potential drug target for overcoming chemotherapeutic resistance. Taken together, these studies establish PCTAIRE1 as a critical mediator of mitotic progression and highlight its role in chemotherapeutic resistance.

This article has an associated First Person interview with the first author of the paper.

## Abstract (precis)

**Summary:** PCTAIRE1 is a mitotic kinase that localizes to the centrosomes, spindle poles and midbody during mitosis, and can serve as a potential drug target to overcome chemotherapeutic resistance.

## INTRODUCTION

PCTAIRE1, also known as cyclin dependent kinase 16 (CDK16 or PCTK1) (Malumbres et al., 2009), belongs to the poorly characterized subfamily of cyclin dependent kinases (CDKs), which includes PCTAIRE1–3 (Manning et al., 2002). These are serine-threonine kinases and are so named based on a specific and conserved sequence of amino acids, i.e. the ‘PCTAIRE’ sequence. PCTAIRE proteins are composed of three principal domains: a long N-terminal domain and a short C-terminal domain, which are unique to each isoform, and a central kinase domain, which is highly conserved between the three isoforms as well as among other CDK members (Cole, 2009). The determination of the crystal structure of PCTAIRE1 kinase domain in association with inhibitors like indirubin-E804 and rebastinib has provided significant insights into the structure of PCTAIRE1 (Dixon-Clarke et al., 2017). PCTAIRE1 is activated by the interaction of the PCTAIRE sequence, present in the α-C helix region of the kinase domain, with the membrane-bound cyclin Y (CCNY) or its homolog cyclin Y like 1 (CCNYL1) (Mikolcevic et al., 2012b; Shehata et al., 2015; Zi et al., 2015). Unlike other CDKs, interaction of cyclin Y with PCTAIRE1 involves regions from the kinase domain as well as a portion of the N-terminal region near the kinase domain.

Although PCTAIRE1 is ubiquitously expressed, its expression is higher in brain, testes and in differentiated cells. PCTAIRE1 is involved in several physiological processes, such as vesicular trafficking, neuronal development, spermatogenesis, glucose homeostasis, and myogenesis (Chen et al., 2012; Liu et al., 2006; Mikolcevic et al., 2012a,b; Mokalled et al., 2010; Palmer et al., 2005; Shehata et al., 2015; Shimizu et al., 2014; Tang et al., 2006; Zi et al., 2015). Our previous research has shown that PCTAIRE1 is regulated by key players in cell proliferation including AKT1, LKB1 (also known as STK11) and BRCA1 (Gillani et al., 2021). Circulating *PCTAIRE1* mRNA has been identified as a biomarker in non-small cell lung cancer (Chang et al., 2020). Interestingly, PCTAIRE1 promotes resistance of prostate and breast cancer cells, but not of the non-transformed cells, to TNF family cytokines by inhibiting the extrinsic apoptotic pathway (Yanagi et al., 2015). Moreover, PCTAIRE1 is overexpressed in numerous cancer cell lines (Charrasse et al., 1999; Yanagi et al., 2014) as well as in some cancers, such as those of skin, liver, prostate and breast (Wang et al., 2017; Yanagi et al., 2017, 2014). Knockdown of PCTAIRE1 decreases the proliferation of several cancer cell lines, including those in cervical, prostate and breast cancers (Yanagi et al., 2017, 2014; Yanagi and Matsuzawa, 2015), and reduces tumor volumes in mouse xenograft models (Wang et al., 2017; Yanagi et al., 2016). The most well-understood role of PCTAIRE1 in the cell cycle is its regulation of p27 (an inhibitor of the CDK4,6-cyclin D complex and a component of the G1/S checkpoint) at serine 10 during S and M phases, followed by its degradation (Yanagi et al., 2017, 2014; Yanagi and Matsuzawa, 2015). Moreover, knockdown of PCTAIRE1 causes growth inhibition and mitotic arrest, which is rescued by silencing p27 (Yanagi et al., 2014).

Polyomavirus small T (PolST) is one of the three early genes that are expressed by the murine polyomavirus genome. Its expression in mammalian cells induces a potent mitotic arrest followed by apoptosis, a phenomenon called ‘mitotic catastrophe’ (Andrabi et al., 2007; Bhat et al., 2020; Pores Fernando et al., 2015). These properties of PolST require its binding to cellular protein phosphatase 2A (PP2A). PP2A is a predominant cellular serine-threonine phosphatase that is composed of three subunits: the structural subunit A, the catalytic subunit C and the regulatory subunit B, thus forming the A-C-B holoenzyme, with each subunit having multiple isoforms. Small T binds PP2A holoenzyme by displacing the associated B subunit from PP2A, and thereby forming the A-C-ST complex, and altering both its localization as well as activity. Because B (B55) and B′ (B56) subunits play a very important role in mitotic regulation in mammalian cells (Grallert et al., 2015; Nilsson, 2018; Wurzenberger and Gerlich, 2011), disruption of the PP2A holoenzyme composition and localization by PolST expression has deleterious consequences on normal mitotic progression of the host cells.

In this study, using PolST as a tool, we screened a library of 196 serine-threonine kinases to identify specific kinases that protect mammalian cells against apoptotic signals that are generated in response to prolonged mitotic arrest as a result of PolST expression. Using this strategy, we identified several candidates that allowed host cells to survive PolST-induced cell death. Among them, PCTAIRE1 consistently offered the best survival phenotype. Hence, we studied PCTAIRE1 in detail to determine its role in mitosis, cell cycle regulation and tumorigenesis. Our results showed that PCTAIRE1 localizes to mitotic spindle and plays an important role during mitosis, including proper spindle and midbody formation. Importantly, we also show that PCTAIRE1 is involved in imparting resistance to cancer cells against antimitotic or chemotherapeutic drugs, thus implying its potential as a drug target in cancer therapy.

## RESULTS

### PCTAIRE1, PLK1, FAK, TAOK3, MAPK12 and GRK5 result in survival against PolST-mediated mitotic arrest and apoptosis

**Fig. 1.**
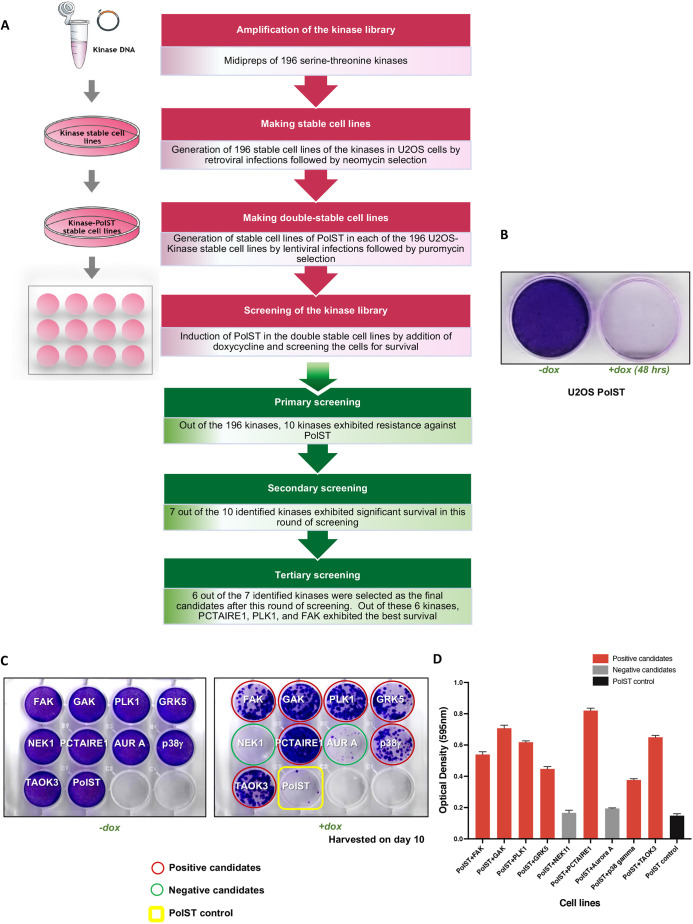
**Screening of serine-threonine kinase library in the presence of PolST expression.** (A) Schematic representation of the workflow for making 196 stable cell lines of kinases and PolST in U2OS background. (B) Crystal Violet staining of PolST-expressing U2OS cells after 48 h of doxycycline induction showing a clear decrease in surviving cells in PolST-expressing cells (+dox) but not in control cells (−dox). (C) Secondary screening of U2OS kinase cell lines in the presence of PolST expression: the kinases that were positive in different rounds of screenings were consolidated in a single plate and subjected to a final and more stringent screening, in which the cells were plated at lower densities. One plate was used as a control for equal plating density (−dox). Cells were allowed to grow for 10 days after doxycycline addition. (D) Graphical representation of the Crystal Violet staining results shown in C. Data are presented as mean±s.d.

A plasmid library of 196 myristoylated serine-threonine kinases, cloned in pWZL-neo-myr-FLAG vector (Addgene) was used to obtain their individual stable cell lines in U2OS background (Fig. 1A; Fig. S1A). This library has been previously used for other screening purposes to identify potential oncogenes, like *IKBKE* (Boehm et al., 2007)*.* We next obtained stable cell lines expressing a particular kinase and PolST using the doxycycline-inducible PolST vector (pTREX-ST-HA-FLAG) (Fig. 1A). After ∼16–20 h of doxycycline addition and hence PolST expression, the cells exhibited mitotic arrest, as was evident by overwhelming cell rounding (not shown), followed by the initiation of apoptosis several hours later, as described previously (Bhat et al., 2020; Pores Fernando et al., 2015). After 48 h of doxycycline addition, most of the PolST-expressing cells underwent cell death, as was shown by Crystal Violet staining (Fig. 1B).

The 196 kinase-PolST double stable cell lines were then monitored for 7−10 days for survival in the presence of PolST expression induced by doxycycline addition (Fig. S1B). Of the 196 kinases, stable cell lines of around ten kinases that exhibited significant survival after this primary screening were further subjected to several rounds of more stringent screenings (Fig. S1C). Because the library constructs had an N-terminal myristoylation tag, we cloned most of these candidates as non-myristoylated constructs in pWZL-blast retroviral vector and obtained their double stable cell lines in PolST background. This was done to rule out the possibility of the kinases leading to survival because of being in a constitutively activated form or being mislocalized to the membranes due to N-terminal myristoylation. After removing the false positives during these screenings, and validating the results using the respective constructs of non-myristoylated kinases, we finally identified six kinases that reproducibly exhibited substantial resistance against PolST-mediated apoptosis, thus resulting in significant survival (Fig. 1C,D; Fig. S1D). These candidates were PCTAIRE1 (CDK16), polo-like kinase 1 (PLK1), focal adhesion kinase (FAK) [also known as protein tyrosine kinase 2 (PTK2)], tao-associated kinase 3 (TAOK3), p38γ (MAPK12) and G protein-coupled receptor kinase 5 (GRK5). Cyclin G-associated kinase (GAK), although consistently scoring positive in most of our screens, was excluded from the final selected candidates as its non-myristoylated version was not able to protect cells from PolST-induced cell death. Among the positive candidates, PCTAIRE1 and PLK1 reproducibly showed very high survival, and therefore, they were taken for further studies.

### PCTAIRE1 allows PolST-expressing cells to exit mitosis

**Fig. 2.**
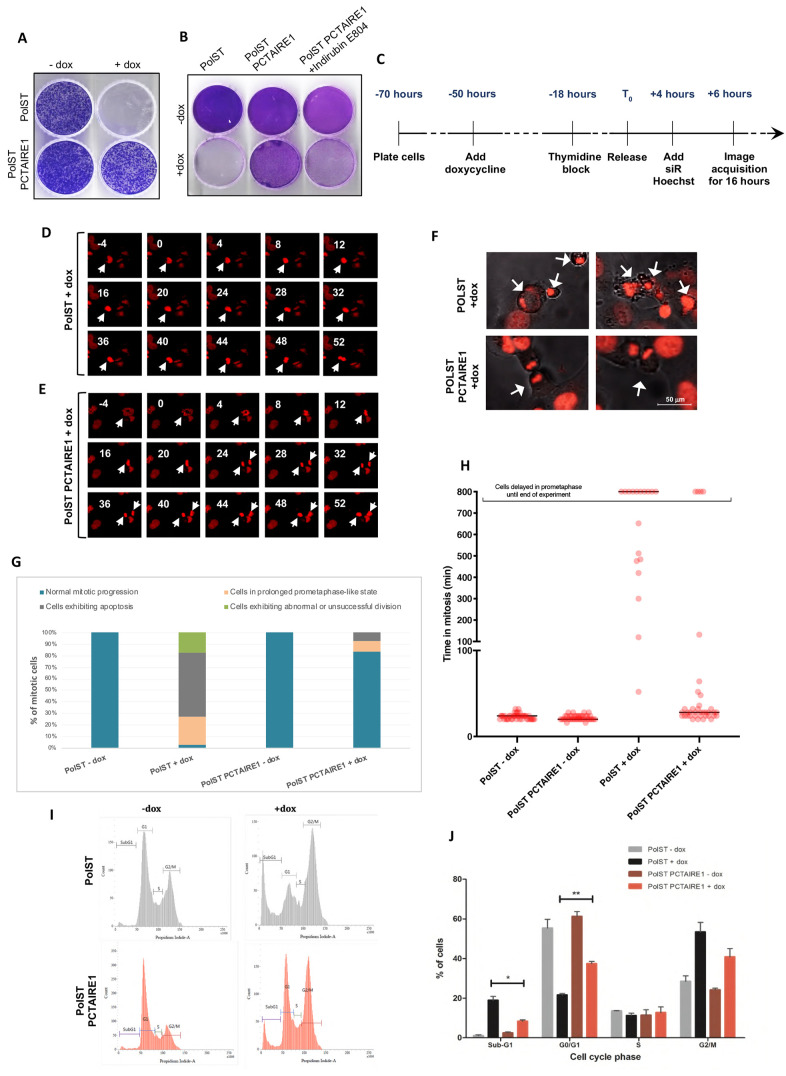
**Overexpression of PCTAIRE1 resists PolST-mediated mitotic arrest and cell death.** (A) Crystal Violet staining showing PCTAIRE1-overexpressing U2OS cells exhibiting higher survival in the presence of PolST expression. Cells were stained after 7 days of doxycycline addition. (B) PCTAIRE1-overexpressing U2OS (PolST-PCTAIRE1) cells were sensitive to PolST-mediated cell death in the presence of PCTAIRE1 inhibitor, indirubin-E804 (500 nM). Cells were stained with Crystal Violet after 7 days. (C) Timeline showing the experimental strategy for live-cell time-lapse imaging for cells in which PolST was induced for 72 h (PolST was induced for 56 h before imaging and the cells were then monitored for 16 h). (D) Examples of time-lapse sequences illustrating the fate of the nucleus of a single representative cell (stained by siR-Hoechst) in the presence of PolST expression (arrows indicate a reference cell during mitosis). (E) Examples of time-lapse sequences illustrating the fate of the nucleus of a single cell (stained by siR-Hoechst) in the presence of both PolST and PCTAIRE1 expression. Numbers indicate minutes with relation to nuclear envelope breakdown (NEBD), which is denoted as 0 min (arrows indicate a reference cell during mitosis). The images in D and E depict areas of 152.18 × 133.16 μm. (F) Representative still images from live-cell time-lapse imaging of PolST cells in U2OS background (72 h induction), showing many mitotically arrested and apoptotic cells in the controls, whereas in the presence of PCTAIRE1 overexpression, there are many cells exhibiting normal cell division (cell structures and the nuclei can be seen when monitored under both differential interference contrast and fluorescence channels; arrows indicate reference cells). (G) Live-cell imaging analysis of PolST cells in U2OS background (in the presence and absence of doxycycline for 72 h), showing PCTAIRE1 overexpression leading to a significant percentage of cells exhibiting normal mitotic progression and decreased apoptosis (the percentage of cells not undergoing mitosis is not shown). Cells in prometaphase-like stage include the cells that were delayed in prometaphase for at least 800 min until the end of the experiment. (Time taken from NEBD to anaphase onset was considered to calculate the duration of mitosis. NEBD was observed by chromosomal condensation and appearance of irregular nuclei, whereas anaphase onset was observed by separation of the two chromosome masses.) (H) Dot plot analysis after live-cell imaging of PolST cells in U2OS background (in the presence and absence of doxycycline for 72 h), showing the time taken for cells in mitosis. Forty mitotic cells were included from each group for the analysis, and the cells undergoing death are not represented in the analysis. The cells that were delayed in a prometaphase-like stage for a minimum of 800 min until the end of the experiment have been categorized separately. (I) FACS analysis of PolST cells in U2OS background (in the presence and absence of PolST for 48 h), showing a decreased population of cells in the sub-G1 and G2/M phases in the presence of PCTAIRE1 overexpression, with a concomitant increase in the G0/G1 population. (J) Graphical representation of the FACS data obtained as in F. Data are presented as mean±s.d.; *P<0.05, **P<0.01 (two-tailed unpaired Student's *t*-test).

Given the library screening results, we validated that cells overexpressing PCTAIRE1 exhibited significantly increased survival in the presence of PolST as compared to the control cells (Fig. 2A). Addition of indirubin-E804, an inhibitor of PCTAIRE1, to these cells for 72 h reversed this survival trend (Fig. 2B). Immunofluorescence studies indicated that all the surviving cells (PolST-PCTAIRE1) had good expression of both PCTAIRE1 as well as PolST, and showed better morphology than the control cells expressing only PolST (Fig. S2). Notably, PCTAIRE1-overexpressing PolST cells had a lower percentage of mitotic/condensed nuclei as shown by 4′,6-diamidino-2-phenylindole (DAPI) staining, and PCTAIRE1 consistently scored as the most impressive candidate from the kinase library in offering survival against PolST-induced cell death; therefore, we decided to predominantly focus on this protein.

Live-cell imaging of PCTAIRE1-overexpressing PolST cells revealed that PCTAIRE1 allowed PolST cells to exit mitosis rather normally, as was indicated by a successful cell division, i.e. separation of the daughter chromosomal sets at anaphase, followed by cytokinesis. This was concomitant with a significant decrease in the number of apoptotic cells. We carried out the live-cell imaging repeatedly under different conditions (i.e. 24, 48 or 72 h of PolST expression) in synchronized cells. The effect of PCTAIRE1 was most prominent in cells induced for 72 h (the cells were monitored for the last 16 h of this period) (Fig. 2C). At this time point, the majority of PolST cells were arrested in a prometaphase-like stage for prolonged periods of time, as is represented by the time-lapse imaging of a PolST-overexpressing cell (Fig. 2D). Most of the cells were not able to reach metaphase at least until 52 min after nuclear envelope breakdown (NEBD), as can be seen in the time-lapse imaging of the represented cell (Fig. 2D). In contrast, most of the PolST cells overexpressing PCTAIRE1 were able to divide successfully. In the time-lapse imaging of the PolST-PCTAIRE1 cell shown in Fig. 2E, at ∼24 min after NEBD, the cell had entered anaphase (Fig. 2E) and was successfully decondensing chromatin and forming daughter nuclei by 52 min. In addition, the arrested PolST cells clearly showed apoptotic features such as membrane blebbing and nuclear fragmentation, whereas the PolST-PCTAIRE1 cells mostly divided successfully (Fig. 2F). These results are shown graphically in Fig. 2G,H, and the movies of the live-cell imaging have been provided in Movies 1–4.

Next, we wanted to study whether PCTAIRE1 has any role in affecting the cell cycle distribution of these cells. Fluorescence-activated cell sorting (FACS) analysis of PolST-PCTAIRE1 cells (+dox) showed a marked decrease in sub-G1 population, concomitant with a drastic increase in the percentage of cells in G0/G1 phases, compared to PolST cells (+dox). Further, the percentage of cells in G2/M phase was lower among PolST-PCTAIRE1 cells (+dox) than among PolST cells. These results were consistent with the live-cell imaging results mentioned above, thus providing strong evidence that PCTAIRE1 overexpression enables PolST cells to successfully exit mitosis and complete cell division, in addition to preventing apoptosis of the cells (Fig. 2I,J).

### PolST-expressing cells exhibit normal mitosis in the presence of PLK1

**Fig. 3.**
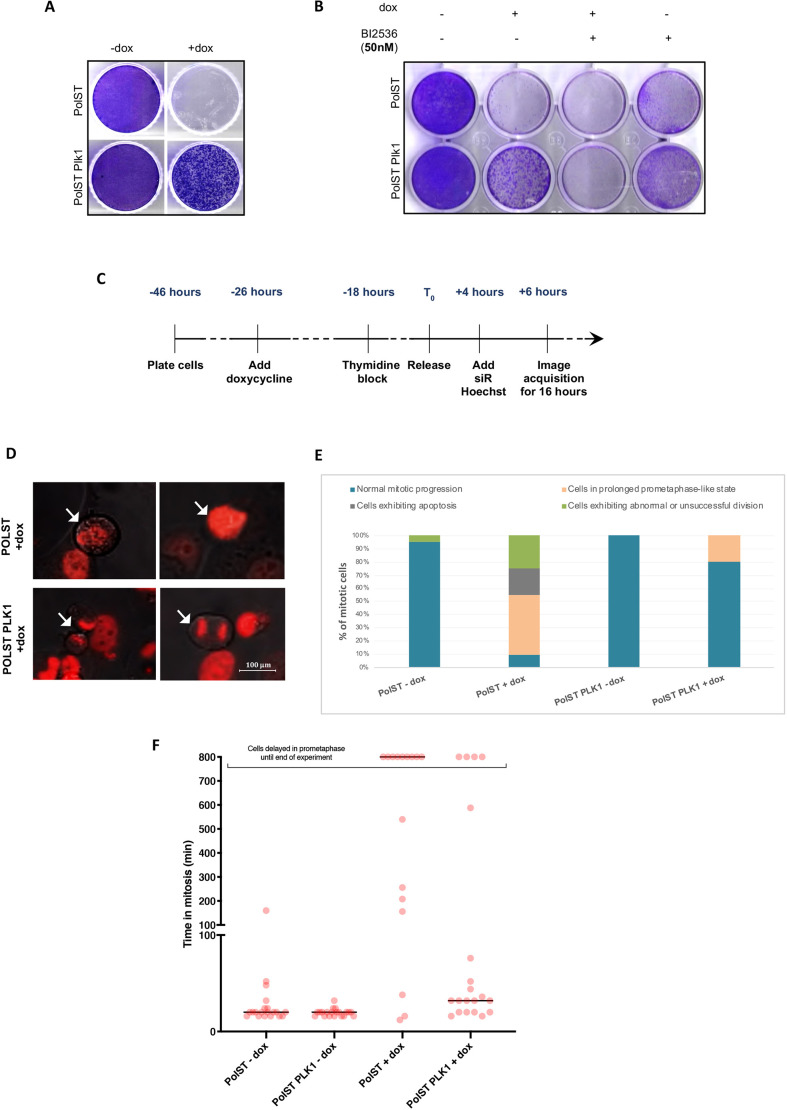
**PLK1 imparts survival against PolST-mediated cell death.** (A) Crystal Violet staining showing enhanced survival of the PLK1-overexpressing stable cell line in U2OS background in the presence of PolST expression. (B) The survival exhibited by PLK1 to PolST-induced cell death was reversed in the presence of PLK1 inhibitor BI-2536 (50 nM). Cells were stained after 1 week. (C) Timeline showing the experimental strategy for live-cell time-lapse imaging for cells in which PolST was induced for 48 h (PolST was induced for 32 h before imaging and the cells were then monitored for 16 h). (D) Representative still images from live-cell time-lapse imaging of PLK1-overexpressing U2OS cells in the presence of PolST (48 h induction), showing some cells dividing successfully in comparison to the controls (arrows indicate reference cells). (E) Graphical representation of the live-cell imaging analysis: PLK1-overexpressing U2OS cells in the presence of PolST (48 h induction) showed a lower percentage of apoptotic cells in comparison to the control cells (the percentage of cells not undergoing mitosis is not shown). Cells in prometaphase-like stage include the cells that were delayed in prometaphase for at least 800 min until the end of experiment. (Time taken from NEBD to anaphase onset was considered to calculate the duration of mitosis. NEBD was observed by chromosomal condensation and appearance of irregular nuclei, whereas anaphase onset was observed by separation of the two chromosome masses.) (F) Dot plot analysis after live-cell imaging of PolST cells in U2OS background (in the presence and absence of doxycycline for 48 h), showing the time taken for cells in mitosis. Twenty mitotic cells were included from each group for the analysis, and the cells undergoing death are not represented in the analysis. The cells that were delayed in a prometaphase-like stage for a minimum of 800 min until the end of the experiment have been categorized separately.

Consistent with our screening results, we observed that PLK1-overexpressing PolST cells also showed substantial survival upon PolST induction (Fig. 3A), which was reversed in the presence of the PLK1 inhibitor BI-2536 (Fig. 3B). The growth inhibition in the presence of both PolST expression and BI-2536 was much stronger than by either of them individually, indicating the role of PLK1 in promoting the survival of cells.

Immunofluorescence results confirmed the expression of both PolST and PLK1 in these cell lines (Fig. S3). As for PCTAIRE1, the PolST-PLK1 cells had healthier morphology than the control PolST cells, thus indicating that PLK1 allowed PolST cells to survive. Additionally, live-cell imaging of the synchronized PolST and PolST-PLK1 cells was also conducted for different time intervals (i.e. 24 or 48 h of PolST expression), and the cells were monitored during the last 16 h of the induction period. In cells induced for 48 h (Fig. 3C), although most of the PolST cells exhibited mitotic arrest and apoptosis, there was a significant decrease in the apoptotic population in PolST-PLK1 cells (+dox). Further, a greater number of cells exhibited normal mitosis among PLK1-overexpressing PolST cells (as indicated by chromosomal separation in anaphase, followed by cytokinesis) than among the control cells, consistent with the increased survival (Fig. 3D). These results are shown graphically in Fig. 3E,F, and the movies of the live-cell imaging have been provided in Movies 5–8.

### PCTAIRE1 provides resistance to cells against chemotherapeutic drugs

**Fig. 4.**
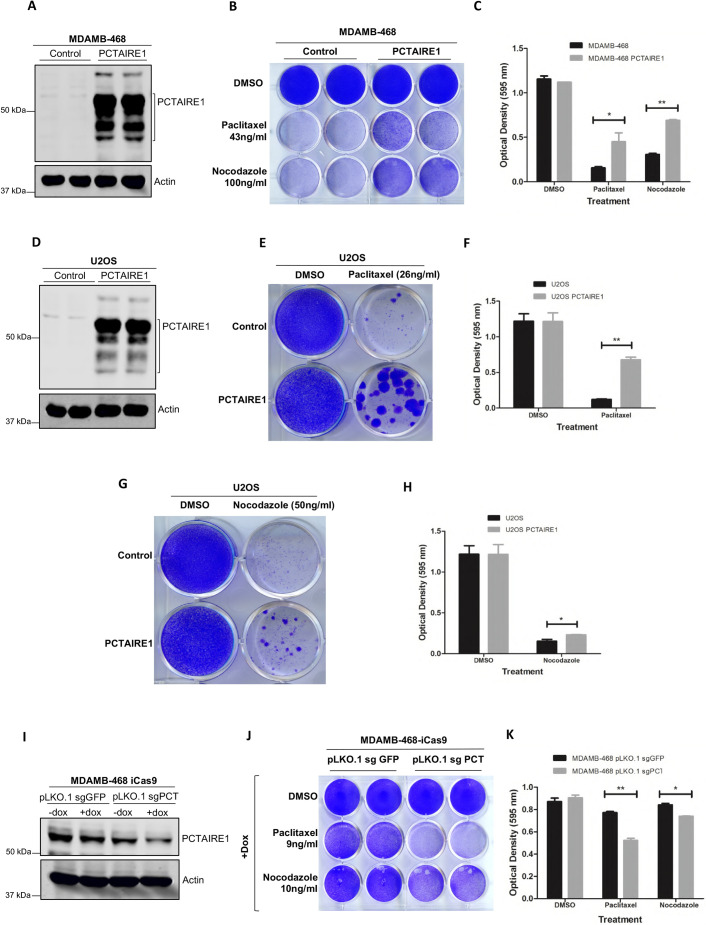
**Expression of PCTAIRE1 promotes resistance against paclitaxel-induced cell death.** (A) Western blot showing confirmation of PCTAIRE1 overexpression cell lines in MDA-MB-468 background. (B) Crystal Violet staining of the PCTAIRE1-overexpressing MDA-MB-468 cell line in the presence of paclitaxel (43 ng/ml) and nocodazole (100 ng/ml), showing increased cell survival compared to the control MDA-MB-468 cells. Cells were harvested after 2 weeks of drug treatments. (C) Graphical representation of the Crystal Violet staining results shown in B. (D) Western blot showing confirmation of PCTAIRE1 overexpression cell lines in U2OS background. (E) Crystal Violet staining of PCTAIRE1-overexpressing U2OS cell lines in the presence of paclitaxel (26 ng/ml), showing a number of surviving colonies of cells compared to the controls. Cells were stained after 2 weeks of drug treatment. (F) Graphical representation of the Crystal Violet staining results shown in E. (G) Crystal Violet staining of PCTAIRE1-overexpressing U2OS cell lines in the presence of nocodazole (50 ng/ml), showing larger and increased number of foci compared to the controls. Cells were harvested after 2 weeks of drug treatment. (H) Graphical representation of the Crystal Violet staining results shown in G. (I) Western blot showing CRISPR/Cas9-mediated knockdown of PCTAIRE1 in MDA-MB-468 cells stably expressing inducible Cas9 (iCas9) by using the sgRNA sequence of PCTAIRE1 indicated as sgPCT4. pLKO.1-sgGFP was used as a control. (J) Crystal Violet staining of PCTAIRE1 knockdown MDA-MB-468 cell lines (pLKO.1-sgPCT4) in the presence of paclitaxel (9 ng/ml) and nocodazole (10 ng/ml), showing decreased cell survival compared to the controls. Cells were harvested after 4 days of drug treatment. (K) Graphical analysis of the Crystal Violet staining results shown in J. Experiments for this figure were repeated 2−3 times. Data are presented as mean±s.d.; *P<0.05, **P<0.01 (two-tailed unpaired Student's *t*-test).

Next, we asked whether PCTAIRE1 also imparts resistance against antimitotic agents that are used in anticancer therapies. We made overexpression cell lines for PCTAIRE1 in two different backgrounds, MDA-MB-468 and U2OS (Fig. 4A,D), which were then treated with antimitotic drugs, paclitaxel and nocodazole. PCTAIRE1-overexpressing cell lines exhibited higher resistance to paclitaxel and nocodazole treatments and showed higher survival in comparison to the controls (Fig. 4B,C,E–H).

To validate these results further, we checked whether targeting PCTAIRE1 would increase their sensitivity to the chemotherapeutic drugs and decrease the apoptotic resistance otherwise exhibited by the cells. CRISPR/Cas9-mediated knockdown of PCTAIRE1 was performed in MDA-MB-468 cells. Stable cell lines of *PCTAIRE1* sgRNAs were made in MDA-MB-468 cells expressing inducible Cas9 (iCas9) (Fig. 4I) and treated with different concentrations of paclitaxel and nocodazole. The results showed that PCTAIRE1-knockdown cells were more sensitive than control cells to paclitaxel and nocodazole treatments (Fig. 4J,K).

### PCTAIRE1 protein levels fluctuate during cell cycle progression

**Fig. 5.**
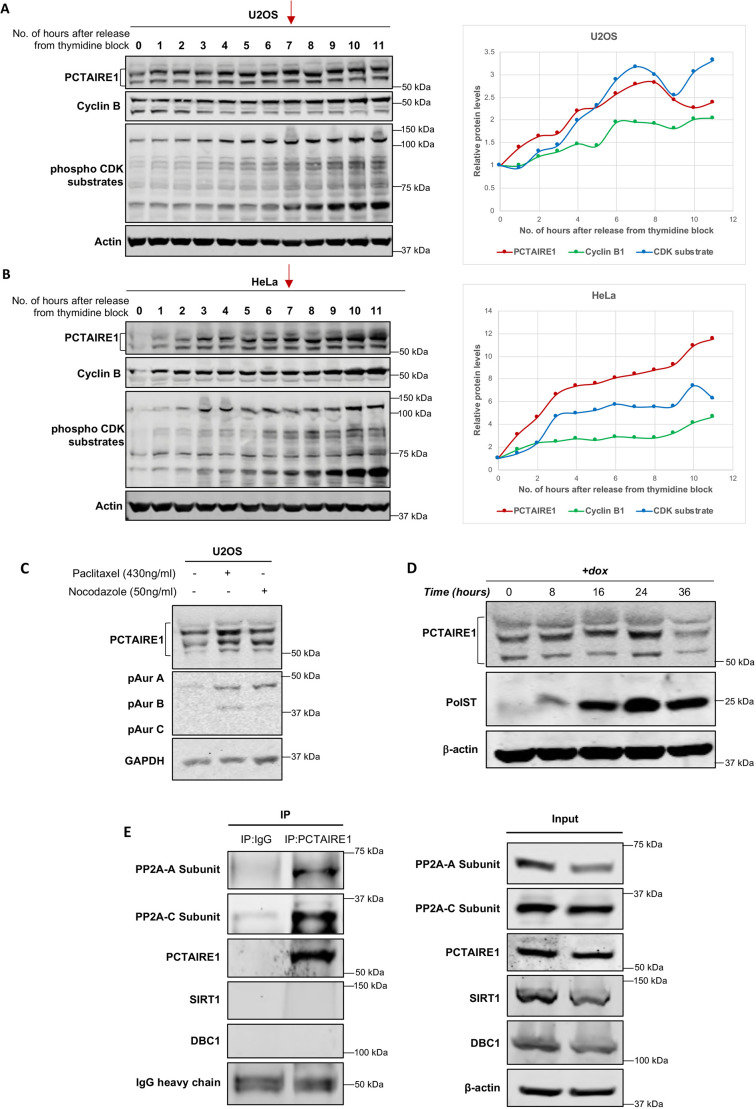
**PCTAIRE1 protein levels change during cell cycle progression.** (A,B) Western blots showing increased PCTAIRE1 protein amounts and phosphorylation levels, as indicated by reduced band mobilities in U2OS (A) and HeLa cells (B), as the cells progressed towards mitosis after release from thymidine block. Mitotic stage was apparent at ∼6–7 h (as shown by the red arrows) after thymidine release, as was confirmed by enhanced phospho-CDK substrate and cyclin B signals. (C) Western blot showing increase in endogenous PCTAIRE1 levels in U2OS cells after the addition of nocodazole (50 ng/ml) and paclitaxel (430 ng/ml) for 24 h. (D) Endogenous PCTAIRE1 levels increased in PolST stable cell lines with increasing time of PolST expression by doxycycline induction up to 24 h. Thereafter, PCTAIRE1 levels decreased sharply. PolST and PCTAIRE1 were detected by anti-HA and anti-PCTAIRE1 antibodies, respectively. (E) Endogenous PCTAIRE1 was pulled down in 293T cells by anti-PCTAIRE1 antibody. A and C subunits of PP2A were co-immunoprecipitated along with PCTAIRE1, as detected by anti-PP2A-A and anti-PP2A-C subunit antibodies, respectively. SIRT1 and DBC1 were used as negative controls for interaction with PCTAIRE1.

PCTAIRE1 belongs to the family of CDKs, which play important roles during cell cycle progression. It is also known to degrade p27 and hence promote the progression of cells through S and M phases (Yanagi et al., 2017). Therefore, we next wanted to check whether PCTAIRE1 protein levels are affected during the cell cycle. Western blot analysis of synchronized U2OS and HeLa cell extracts showed that, as the cells progressed through S phase, PCTAIRE1 levels steadily increased and peaked during mitosis (Fig. 5A,B). This was also associated with reduced band mobilities of PCTAIRE1 (at ∼6–7 h post release), which indicated a possible increase in the phosphorylation levels of PCTAIRE1, and this was more appreciably seen in Fig. S4A,B. Concomitant enhanced phosphorylation of CDK substrates confirmed that the cells were in mitotic phase at this time period. To further validate that the cells were in mitosis during this time period, we also performed phospho-histone 3 (Ser10) staining on the cells (Fig. S4C–E). Western blotting confirmed that, with an increase in the percentage of mitotic index (as shown by phospho-histone 3 staining), there was a concomitant increase in the protein levels of PCTAIRE1 as well as of known mitotic markers such as cyclin B1 and phospho-CDK substrates.

To further validate that PCTAIRE1 amounts increase during mitosis, we arrested U2OS cells in mitosis using paclitaxel and nocodazole. Interestingly, endogenous PCTAIRE1 protein levels were also enhanced in these mitotically arrested cells (Fig. 5C). Similar increases in PCTAIRE1 levels were also seen in PCTAIRE1-overexpressing U2OS stable cells when treated with nocodazole (Fig. S5A). A similar time-dependent increase in endogenous PCTAIRE1 levels, as well as its phosphorylation status (as indicated by band shift), was also noticed in cells that were arrested in mitosis by PolST (Fig. 5D). However, at 36 h of PolST expression, there was a sudden decrease in PCTAIRE1 amounts, which could be attributed to severe apoptosis occurring in these cells following chronic mitotic arrest. We have also seen similar trends for sudden decrease in the levels of some other proteins, like UNC5B, after 24 h of PolST expression (Bhat et al., 2020). Further, as with the endogenous PCTAIRE1 levels until 24 h of PolST expression, we observed a similar stabilization of exogenously expressed PCTAIRE1 levels when cells were co-transfected with PolST (Fig. S5B).

Because mitotic arrest and apoptosis induced by PolST require its binding to PP2A (Andrabi et al., 2007; Bhat et al., 2020; Pores Fernando et al., 2015), we reasoned that PCTAIRE1 might be overcoming the effects of PolST by somehow affecting PP2A. To test this possibility, we checked whether PCTAIRE1 interacts with PP2A. Pulldown of endogenous PCTAIRE1 in 293T cells showed that both the A and C subunits of PP2A were co-immunoprecipitated with PCTAIRE1 (Fig. 5E), thus confirming that PCTAIRE1 interacts with PP2A. To validate that this interaction of PP2A subunits is specific to PCTAIRE1, we also probed DBC1 (also known as CCAR2) and SIRT1 in the same experiment. This was relevant, as our other studies have shown that PolST interacts with DBC1, which consequently affects its association with SIRT1 deacetylase (Sarwar et al., 2022). Our results did not show any such interaction between PCTAIRE1 and these proteins, thus confirming that the interaction between PCTAIRE1 and PP2A subunits is specific.

The above results thus showed that PCTAIRE1 has a role in mitotic progression, as it overcomes mitotic arrest and also interacts with PP2A, a critical regulator of mitosis. However, to check whether overexpression of PCTAIRE1 also promotes mitotic progression, we performed immunofluorescence in PCTAIRE1-overexpressing cells using phospho-histone 3 staining. Results indeed showed an increase in the mitotic index in PCTAIRE1-overexpressing cells, with a concomitant increase in the levels of cyclin B1 and phospho-histone 3 (Fig. S6A–C). Conversely, the levels of phospho-CDK substrates and phospho-histone 3 were decreased upon PCTAIRE1 knockdown in cells using endoribonuclease-prepared siRNA (esiRNA) against *PCTAIRE1* (Fig. S6D).

### PCTAIRE1 interacts with PLK1

**Fig. 6.**
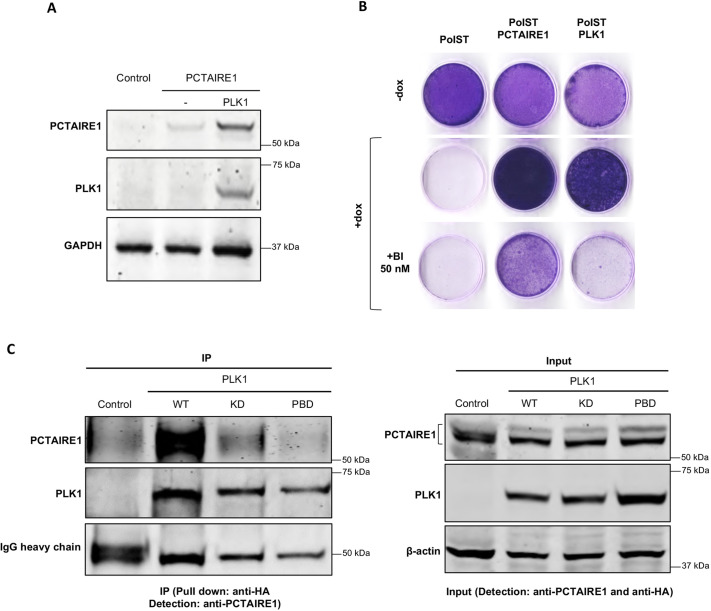
**PCTAIRE1 interacts with PLK1.** (A) After transient transfections of 1 µg each of FLAG-PCTAIRE1 and myc-PLK1 in 293T cells, PCTAIRE1 was stabilized. (B) Crystal Violet staining of U2OS-PolST cells showing reversal of cell survival in the presence of PLK1 inhibitor BI-2536 (50 nM) in PLK1-overexpressing PolST (PolST-PLK1) cells but not in PCTAIRE1-overexpressing PolST (PolST-PCTAIRE1) cells. (C) HA-tagged constructs of wild-type PLK1 (WT), PLK1 kinase-dead mutant (KD) and PLK1 polo-box domain mutant (PBD) were transiently transfected in U2OS cells (1 µg each). After pulling down PLK1 by anti-HA antibody, PCTAIRE1 was co-immunoprecipitated with WT-PLK1 but not with KD and PBD mutants.

Because PLK1, another identified kinase in our screenings, is already well-known to promote key mitotic events, we wanted to test whether PLK1 also has any impact on PCTAIRE1. Interestingly, when co-transfected with PLK1 in 293T cells, PCTAIRE1 was stabilized (Fig. 6A). Similar results were obtained when cells were subjected to synchronization by thymidine block for 18 h and harvested either immediately (interphase) or after an additional 7 h (approximately at G2 or M) (Fig. S7A,B).

In light of the above results, we wanted to investigate whether PLK1 could be acting upstream of PCTAIRE1 to promote the survival of PolST-expressing cells. Therefore, we tested whether PCTAIRE1 expression could restore cell growth in the presence of the PLK1 inhibitor BI-2536 (Fig. 6B). Strikingly, BI-2536 fully reversed the pro-survival effect of PLK1, but only partially inhibited cell growth promoted by PCTAIRE1. This finding is consistent with the possibility that PLK1 acts, at least in part, through upregulating PCTAIRE1 to allow growth of PolST-expressing cells.

To study the link between PLK1 and PCTAIRE1 further, we checked whether the two proteins interact with each other. We pulled down endogenous PCTAIRE1 from cells that were transfected with either wild-type PLK1 or with its mutant versions, i.e. PLK1 kinase-dead and PLK1 polo-box domain mutants. Results showed that PCTAIRE1 was co-immunoprecipitated with wild-type PLK1 but not with the kinase-dead and polo-box domain mutants (Fig. 6C).

### PCTAIRE1 localizes to the centrosomes, spindle poles, central spindle and midbody during mitosis

**Fig. 7.**
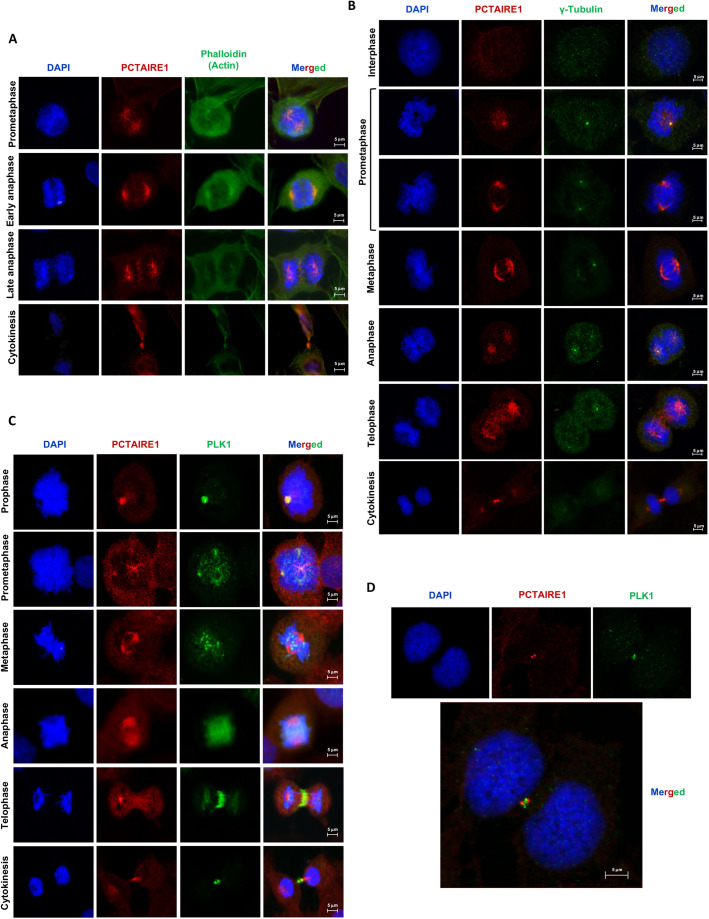
**Localization of PCTAIRE1 in different phases of cell cycle.** (A) Immunofluorescence studies of U2OS cells using actin binding dye, phalloidin and anti-PCTAIRE1 antibody. During prometaphase and metaphase, PCTAIRE1 can be seen on the mitotic spindle, including the spindle poles. At anaphase and cytokinesis, it is also seen at the midplate and midbody, respectively. DAPI was used for chromosomal staining to identify a particular phase of mitosis. (B) Staining of U2OS cells by anti-γ-tubulin and anti-PCTAIRE1 antibodies showing that PCTAIRE1 colocalizes with γ-tubulin in the centrosomes and at spindle poles throughout mitosis (63× magnification). (C) PCTAIRE1 and PLK1 localizations during mitosis in U2OS cells: PCTAIRE1 colocalizes with PLK1 at centrosomes (prophase), spindle poles (prometaphase) and then later at the midbody (telophase, cytokinesis) (63× magnification), but not at metaphase and anaphase stages. (D) PCTAIRE1 localizes just adjacent to PLK1 at the midbody (63× magnification).

Immunofluorescence studies on U2OS cells in the presence of phalloidin (for staining actin) revealed that PCTAIRE1 is present in the cytoplasm as well as in the nucleus during interphase (data not shown). However, as the cells progress towards late G2 and M phases, PCTAIRE1 can be observed predominantly at the spindle poles, as well as on the microtubules. As the cells move from anaphase to cytokinesis, PCTAIRE1 is observed prominently at the central spindle and midbody (Fig. 7A). Using immunostaining against γ-tubulin, we further confirmed that PCTAIRE1 localizes to the centrosomes (Fig. 7B). Because PCTAIRE1 was seen to localize on the spindle as well as at the poles, we wanted to check its localization with respect to the well-known mitotic kinase PLK1, which was also identified as a prominent candidate in our screenings (Fig. 1C). This was also relevant as we had observed that PCTAIRE1 amounts are stabilized in the presence of PLK1 (Fig. 6A), and PLK1 was also seen to interact with PCTAIRE1 (Fig. 6C). Immunofluorescence results showed that, during prophase and pro-metaphase, PLK1 colocalizes with PCTAIRE1 at centrosomes and at microtubules near the poles (Fig. 7C). However, in metaphase–anaphase, whereas PLK1 was seen mainly around the chromosomes/kinetochores, PCTAIRE1 was still predominantly present towards the poles. Quite interestingly, in telophase, PCTAIRE1 was distributed all along the microtubules of the elongated spindle in the midzone, including the central spindle, whereas PLK1 seemed to be predominantly focused at the central spindle and the contractile ring region (Fig. 7C). During cytokinesis, both PCTAIRE1 and PLK1 were in close proximity (in a crisscross orientation) at the midbody (Fig. 7D).

### PCTAIRE1 is critical for normal mitotic progression

**Fig. 8.**
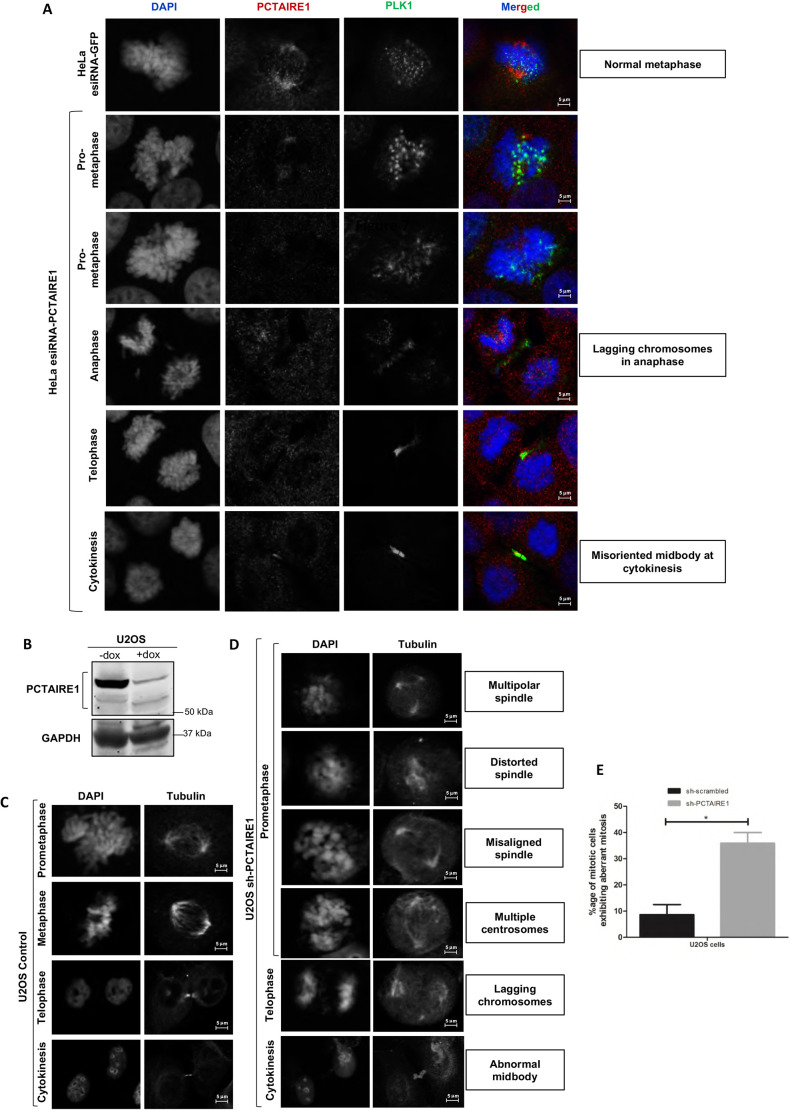
**Knockdown of PCTAIRE1 causes mitotic arrest and apoptosis.** (A) Immunofluorescence of HeLa cells transfected with esiRNA for *PCTAIRE1* and stained 72 h post-transfection showed aberrant mitosis. Many cells exhibited abnormal metaphase, with lagging chromosomes at anaphase and misoriented midbodies during cytokinesis. (B) Western blot analysis confirming knockdown of PCTAIRE1 in U2OS cells by shRNA (after 72 h of shRNA induction using doxycycline). (C) DAPI and tubulin staining in control U2OS cells to detect the spindle and nuclear morphologies during different stages of mitosis. (D) Effect of shRNA for *PCTAIRE1* on mitosis in U2OS cells (after 72 h of shRNA induction using doxycycline): immunofluorescence of PCTAIRE1 knockdown in U2OS cells predominantly led to aberrant mitosis with multipolar, misaligned or distorted spindles, in addition to presence of multiple centrosomes, lagging chromosomes and abnormal midbody, as was observed by tubulin staining. (E) Quantitative analysis of aberrant mitosis in sh-PCTAIRE1 cell lines. Data shown are the mean±s.d. of *n*=3 independent experiments; *P<0.05 (two-tailed unpaired Student's *t*-test). All images were taken at 63× magnification.

In order to check the significance of the role of PCTAIRE1 in mitosis, we performed knockdown of PCTAIRE1 using esiRNA in HeLa cells. PCTAIRE1 knockdown, which was confirmed by immunofluorescence, arrested a significant proportion of cells in mitosis. Further, the majority of such cells had abnormal spindle morphologies and exhibited numerous mitotic abnormalities, such as premature chromosome segregation, lagging chromosomes in anaphase and abnormally oriented midbodies between the daughter cells during cytokinesis (Fig. 8A).

In order to further confirm these results, we obtained an inducible pLKO.1-PCTAIRE1 shRNA stable cell line in U2OS cells and confirmed the knockdown by western blotting (Fig. 8B). In comparison to control cells (Fig. 8C), a substantial percentage of PCTAIRE1-knockdown cells appeared delayed at a prometaphase-like stage or exhibited aberrant mitosis (Fig. 8D,E). These abnormalities included multiple centrosomes, multipolar spindles and lack of proper spindle formation, thus implying the importance of PCTAIRE1 in proper mitotic progression. These results were observed in LN18 cells as well (Fig. S8).

## DISCUSSION

Using the doxycycline-regulated PolST-expressing cell line, we set out to screen a library of serine-threonine kinases to identify the candidates that would enable cells to survive against PolST-mediated cell death. Among the six positive candidates, PCTAIRE1 reproducibly showed the most prominent survival phenotype. These results were obtained with both myristoylated as well as the non-myristoylated versions of most of these kinases.

The survival potential imparted by PCTAIRE1 was, however, not just restricted to that against PolST, as PCTAIRE1 was also able to rescue cells from mitotic arrest/apoptosis caused by antimitotic drugs like paclitaxel, a conventional chemotherapeutic drug commonly used for cancer treatment. Given our results showing that PCTAIRE1 overexpression imparts resistance to cells against paclitaxel, whereas PCTAIRE1 knockdown sensitizes cells to this drug, our findings indicate a potential role of PCTAIRE1 in chemotherapeutic resistance, a possibility that will be interesting to explore in the future.

We have also shown that PCTAIRE1 localizes to mitotic spindle during cell division, which is another interesting novel finding. The localization pattern of PCTAIRE1 changes during different stages of mitosis, as it was seen at the centrosomes during late interphase, at spindle poles during prophase, at polar microtubules during pro-metaphase/metaphase and at the midbody during cytokinesis. In addition, we have shown that PCTAIRE1 protein levels also fluctuate during different stages of the cell cycle, reaching its peak during mitosis. This resembles the pattern of oscillation seen in the case of important mitotic proteins including PLK1, Aurora kinases, and Cyclins A and B, thereby suggesting that, like these mitotic proteins, PCTAIRE1 levels increase as a function of mitosis. We have shown that PLK1 interacts with PCTAIRE1, and we speculate that PCTAIRE1 may be a substrate of PLK1 and could thus be regulated by PLK1-mediated phosphorylation. PLK1 is known to phosphorylate the consensus motif D/E-X-S/T-φ (where X is any amino acid and φ is any hydrophobic amino acid). Interestingly, PCTAIRE1 has two phosphorylation motifs for PLK1 phosphorylation in the α-helix loop of the kinase domain (EVSL 211-214 and EKSL 232-235), which could be potentially phosphorylated by PLK1, and a PLK1 consensus motif at T175 is known to be phosphorylated in PCTAIRE1 (Olsen et al., 2010). However, it remains to be seen whether these sites are the bona fide targets for PLK1. It is therefore quite possible that PLK1 and PCTAIRE1 are players in the same pathway.

Knockdown of PCTAIRE1 by RNA interference generated aberrant mitotic phenotypes such as multipolar spindles and misoriented midbodies in cytokinesis. Therefore, our results suggest that the presence of PCTAIRE1 is critical for cells to exhibit proper mitosis and divide successfully, thus indicating that PCTAIRE1 must play a significant role in mitotic regulation. These findings are consistent with other studies in which PCTAIRE1-knockdown cells were seen to exhibit mitotic arrest (Wang et al., 2017; Yanagi et al., 2014). The ability of PCTAIRE1, when overexpressed, to promote survival when cells are delayed in mitosis by PolST or submaximal concentrations of spindle poisons points to a role for the kinase in allowing cells to extinguish mitotic spindle checkpoint signaling. Because PolST is a known regulator of PP2A (Mullane et al., 1998), and PP2A plays a role in turning off checkpoint signaling (Nilsson, 2018), it is quite possible that PCTAIRE1 acts, at least in part, through modulating PP2A activity. In that case, it would be similar to PLK1 (which was also identified as a positive candidate in our screen), that is already reported to regulate PP2A (Nilsson, 2018). Interestingly, our results have indeed shown that PCTAIRE1 interacts with the A and C subunits of PP2A, thus suggesting a PP2A-dependent mechanism of action of PCTAIRE1 in overcoming mitotic arrest in cells.

We also speculate that PCTAIRE1 particularly plays an important role in cytokinesis. In our studies, PCTAIRE1 was observed prominently at the midbody, where it was seen in close proximity to PLK1. Interestingly, in a high-throughput mass-spectrometry analysis, PCTAIRE1 has recently been reported to interact with PRC1 (Hernández-Ortega et al., 2019). PRC1 is a key modulator of cytokinesis and is known to be regulated by CDK1, PLK1, Aurora kinase B, and phosphatases like PP1 and PP2A (Cundell et al., 2013; Hu et al., 2012; Nunes Bastos et al., 2013). PRC1 plays a very important role in interdigitating microtubules, along with centralspindlin (a heterotetramer of MKLP1, also known as KIF23, and CYK4, also known as MgcRacGAP and RACGAP1), during central spindle formation. In addition, it is reported that PCTAIRE1 phosphorylates p27 at serine 10, promoting its degradation, which would thus promote the entry of cells into S and M phases (Yanagi et al., 2017, 2014; Yanagi and Matsuzawa, 2015). Other studies have also reported a crucial role of p27 in cytokinesis. p27 has been shown to bind to citron kinase (CK), thus preventing the binding of CK to RhoA and inhibiting its ability to stimulate actinomyosin activation and contractile ring formation between the dividing cells (Serres et al., 2012). Therefore, phosphorylation of p27 at serine 10 by PCTAIRE1, followed by its degradation, could promote the interaction between CK and RhoA, thus promoting constriction of the contractile ring and thereby forming the midbody. These studies, together with our results, strongly suggest that PCTAIRE1 is an important component of the midbody machinery. However, further studies are required to elucidate the exact mechanism of action of PCTAIRE1 at cytokinesis as well as in promoting mitotic exit.

Given our results, we believe that PCTAIRE1 can be a potential target for effective anticancer treatment. Various inhibitors against PCTAIRE1 have been developed and tested, with indirubin E-804, rebastinib and dabrafenib being the most promising at inhibiting PCTAIRE1 activity (Dixon-Clarke et al., 2017; Margue et al., 2019; Pophali and Patnaik, 2016). These PCTAIRE1 inhibitors may thus be tested, either by themselves, or in combination with other chemotherapeutic drugs for effective anticancer treatment and for overcoming drug resistance in cancers.

## MATERIALS AND METHODS

### Constructs, reagents and antibodies

An open reading frame (ORF) library of 196 serine-threonine kinases was purchased from Addgene. PCTAIRE1, FAK, GAK, GRK5, p38γ, TAOK3 and PLK1 of the library were subcloned from pWZL-neo-myr-FLAG to pWZL-blast and pCMV-HA-FLAG. shRNA for *PCTAIRE1* was cloned in inducible vector pLKO.1-puromycin (Addgene) using the following oligonucleotides: 5′-CCGGGCTCTCATCACTCCTTCACTTCTCGAGAAGTGAAGGAGTGATGAGAGCTTTTTG-3′ and 5′-AATTCAAAAAGCTCTCATCACTCCTTCACTTCTCGAGAAGTGAAGGAGTGATGAGAGC-3′. iCAS9-neo was obtained from Addgene, and sgRNAs for *PCTAIRE1* were cloned in pLKO.1-puro using the following sequences: sgPCT1, 5′-CACCGCCCACGCAAGATCTCCACTG-3′ and AAACCAGTGGAGATCTTGCGTGGGC; sgPCT2, 5′-CACCGTCAGACTGGAACATGAAGAG-3′ and 5′-AAACCTCTTCATGTTCCAGTCTGAC-3′; sgPCT3, 5′-CACCGGGGCACCCTGCACCGCCATC-3′ and 5′-AAACGATGGCGGTGCAGGGTGCCCC-3′; sgPCT4, 5′-CACCGACTGGAGACCTACATTAAGC-3′ and 5′-AAACGCTTAATGTAGGTCTCCAGTC-3′. esiRNA for *PCTAIRE1* was purchased from Sigma-Aldrich.

The antibodies used included mouse anti-FLAG (Sigma-Aldrich; #F3165; 1:5000 dilution), rabbit anti-FLAG (Cell Signaling Technology; #14793; 1:5000 dilution), mouse anti-HA (Sigma-Aldrich; #26183; 1:5000 dilution), rabbit anti-HA (Cell Signaling Technology; #3724; 1:5000 dilution), rabbit anti-myc tag (Cell Signaling Technology; #2278; 1:1000 dilution), rabbit anti-PCTAIRE1 (Cell Signaling Technology; #4852S; 1:1000 dilution), rabbit anti-PCTAIRE1 (Novus Biologicals; #NBP1-92248; 1:5000 dilution), rabbit anti-PCTAIRE1 (Novus Biologicals; #NBP2-55219; 1:50 dilution for immunofluorescence), mouse anti-β-tubulin (Sigma-Aldrich; #T8328; 1:1000 dilution), rabbit anti-β-tubulin (Cell Signaling Technology; #15115S; 1:1000), rabbit anti-GAPDH (Cell Signaling Technology; #2118; 1:10,000 dilution), mouse anti-vinculin (Cloud clone; #CAB839Hu22; 1:1000 dilution), mouse anti-actin (Cell Signaling Technology; #3700; 1:10,000 dilution), rabbit anti-phospho Aurora A,B,C (Cell Signaling Technology; #2914; 1:1000 dilution), rabbit anti-PLK1 (Abcam; #ab189139; 1:1000 dilution), rabbit anti-phospho-histone 3 (Cell Signaling Technology; #53348; 1:100 dilution for immunofluorescence), rabbit anti-phospho-CDK substrate (Cell Signaling Technology; #14371; 1:1000 dilution), rabbit anti-PP2A-A subunit (Cell Signaling Technology; #2041; 1:1000 dilution), rabbit anti-PP2A-C subunit (Cell Signaling Technology; #2038; 1:1000 dilution), rabbit anti-Cyclin B1 (Cell Signaling Technology; #12231; 1:1000 dilution), rabbit anti-SirT1 (Cell Signaling Technology; #2493; 1:1000 dilution), mouse anti-DBC1 (Cell Signaling Technology; #5857; 1:1000 dilution), DyLight™ 680 Conjugate (Cell Signaling Technology; #5470S; 1:10,000 dilution), DyLight™ 800 4X PEG Conjugate (Cell Signaling Technology; #5151S; 1:10,000 dilution). ProLong glass antifade DAPI was obtained from Invitrogen. siR-Hoechst, thymidine, propidium iodide, polybrene, polyethylenimine (PEI) and doxycycline were purchased from Sigma-Aldrich. Neomycin, puromycin dihydrochloride and blasticidin S hydrochloride were purchased from InvivoGen, Amresco and Invitrogen, respectively. Nocodazole, paclitaxel and indirubin-E804 were purchased from Millipore. Protease and phosphatase inhibitors were purchased from Roche.

### Cell culture

Cells were cultured in Dulbecco's modified Eagle medium (DMEM) (Gibco-Life Technologies) supplemented with 10% fetal bovine serum (FBS; Gibco-Life Technologies) and 1% pencillin-streptomycin (Cell Clone). Cells were split by trypsinization using 0.25% Trypsin-EDTA (Gibco-Life Technologies). Freeze downs of cells were made in freezing medium (FBS+10% DMSO) and stored in liquid nitrogen.

### Making a stable cell line library of kinases and PolST

The kinase ORFs were present in pWZL-neo-myr-FLAG retroviral vector backbone, having myristoylation and FLAG tags. Amplifications of these plasmid DNAs were carried out by Thermo Fisher Scientific Midiprep Kits. Retroviruses encoding specific kinases from the library were produced by transfection of 293T cells (American Type Culture Collection, ATCC) with corresponding retroviral vector DNAs from the library (3 µg) together with the packaging plasmids encoding gag pol (1 µg) and VSV-G (1 µg) using PEI (Sigma-Aldrich). Retroviral titers were collected 48 and 72 h post-transfection. U2OS osteosarcoma cells (ATCC) were infected with the retrovirus in the presence of polybrene (8 µg/ml). Cells were grown for a day post-infection, after which they were trypsinized, centrifuged at 200 ***g*** for 5 min, and seeded at low confluence in fresh culture dishes. The cells were selected in neomycin (600 ng/ml)-containing medium for ∼2 weeks to generate individual stable cell lines for each of the 196 kinases from the library. To obtain stable cell lines expressing the respective kinases together with PolST, we used an inducible lentiviral construct of PolST present in pTREX-puro-HA-FLAG vector backbone. Lentiviruses were generated in 293T cells by transfecting them with PolST DNA (2.5 µg) together with Δ8.9 (2.25 µg) and VSV-G (0.25 µg). The 196 U2OS-kinase stable cell lines were infected with the lentiviral titer of PolST (as described earlier for retroviral infections) and then selected in puromycin (5 µg/ml)-containing medium for ∼3 days. Selected stable cell lines of the kinases in the presence and absence of PolST in U2OS background were amplified for experiments and frozen down with freezing medium in liquid nitrogen for long-term storage.

### CRISPR/Cas9-mediated knockdown of PCTAIRE1

Four sgRNAs for *PCTAIRE1* were cloned in pLKO.1 puro vector. The sequences of the oligonucleotides used were as follows: sgPCT forward-1, 5′-CACCGCCCACGCAAGATCTCCACTG-3′ and sgPCT reverse-1, 5′-AAACCAGTGGAGATCTTGCGTGGGC-3′; sgPCT forward-2, 5′-CACCGTCAGACTGGAACATGAAGAG-3′ and sgPCT reverse-2, 5′-AAACCTCTTCATGTTCCAGTCTGAC-3′; sgPCT forward-3, 5′-CACCGGGGCACCCTGCACCGCCATC-3′ and sgPCT reverse-3, 5′-AAACGATGGCGGTGCAGGGTGCCCC-3′; sgPCT forward-4, 5′-CACCGACTGGAGACCTACATTAAGC-3′ and sgPCT reverse-4, 5′-AAACGCTTAATGTAGGTCTCCAGTC-3′. A two-vector system of iCas9 neo and pLKO.1 puro-sgPCT (1−4) was used to make cell lines of iCas9 neo and *PCTAIRE1* sgRNAs in U2OS and MDA-MB468 cells (ATCC). For this, the cell line of iCas9 was made first and selected using neomycin, and then the cell line of the pLKO.1 sgRNAs was made in the same background using puromycin selection.

### Clonogenic assay

Cells were seeded in 12-well plates (5×10^3^ to 10×10^3^ cells per well) and allowed to grow overnight in regular growth medium. Cells were then grown in the presence or absence of drug (as indicated) for 1−2 weeks, during which their medium was replaced every alternate day. Cells were then fixed with methanol for 10 min, stained with 0.5% Crystal Violet and photographed using a digital scanner.

### Western blotting and immunoprecipitation

For western blotting, cells were lysed with the requisite amounts of lysis buffer (NP-40) supplemented with freshly prepared phosphatase and protease inhibitors (Complete, Roche), and centrifuged at 13,000 ***g*** at 4°C for 10 min. Then, 5× sample buffer was added to the supernatants, and the samples were boiled at 100°C for 5 min. Standard protocol was followed to run protein samples on SDS-PAGE, and they were transferred to nitrocellulose membrane using Mini Trans-Blot^®^ apparatus (Bio-Rad). The blot was blocked in 3% bovine serum albumin (BSA) for 1 h and incubated overnight with the requisite primary antibody. After washing twice with TBST (TBS containing 0.05% Tween-20) (for 15 min each) and once with TBS (for 10 min), the blot was incubated with the corresponding secondary antibodies for 1 h. This was again followed by washing the blot in TBST and TBS as earlier, and the blot was then detected using a Bio-Rad Chemidoc MP system (after incubation with chemiluminescence substrate for 5 min) or a Li-Cor Odyssey system.

For immunoprecipitation, protein A or G sepharose beads were incubated with the requisite antibody for pulldown. These antibody-conjugated beads were then added to the protein supernatants obtained after cell lysis (as mentioned above) and incubated overnight at 4°C. The beads were pelleted down and washed three times with PBS, after which 2× sample buffer was added to them. The samples were then boiled at 100°C for 5 min and subjected to western blotting as described above.

### Live-cell imaging

Cells were seeded in four-chambered live-cell imaging dishes (Greiner Bio One). siR-Hoechst DNA-binding dye and Verapamil were then added to the cells along with fresh media at dilutions of 0.25 µM and 10 µM, respectively, and incubated for 2 h. Time-lapse imaging was then carried out at 37°C, 5% CO_2_ for ∼16 h using an inverted Nikon A1R confocal microscope (10× magnification). *Z*-stack images of 12 sections, each 1 µm apart, were captured approximately every 4 min. Movies were then analyzed using Nikon imaging software (NIS Elements Viewer).

### Immunofluorescence

Cells were seeded in culture dishes containing poly-L-lysine-coated glass cover slips and allowed to grow under required conditions. After washing with PBS, the cells were fixed with 4% formaldehyde for 10 min in the dark. The cells were washed three times with PBS and permeabilized with 0.2% Triton X-100 (in PBS). Next, the cells were blocked with 2% BSA (in PBS) for 1 h. The cover slips were then incubated with the corresponding primary antibody at 37°C for 3−4 h in a moisture chamber. After washing the coverslips twice with PBST (PBS, 0.05% Tween 20) and once with PBS, they were incubated with corresponding secondary antibodies for 1 h in a moisture chamber as mentioned above. The coverslips were again washed twice with PBST and once with PBS, and then mounted on glass slides with ProLong glass antifade DAPI solution and observed under a microscope.

### FACS

For cell cycle analysis by flow cytometry, cells were grown to a confluence of ∼50−60% in 60 mm dishes. At the time of fixing, cells were washed once with PBS and then incubated with PBS supplemented with 0.1% EDTA for 5 min at 37°C. Cells were centrifuged at 1000 ***g*** for 5 min, washed with PBS supplemented with 1% serum, and centrifuged again at 1000 ***g*** for 5 min. The pellets were resuspended in 500 μl PBS and fixed by the addition of 5 ml ethanol to the tubes in a drop-wise manner while vortexing. For subjecting the samples to flow cytometry, the samples were centrifuged at 1000 ***g*** for 5 min, and cell pellets were washed once with 1% serum (in PBS) and centrifuged at 1000 ***g*** for 5 min. The cell pellets were then resuspended in 200−500 µl propidium iodide/RNase solution (50 µg/ml propidium iodide, 10 mM Tris-HCl, pH 7.5, 5 mM MgCl_2_ and 20 mg/ml RNase A). After incubating the samples at 37°C for 30 min, they were subjected to flow cytometry using BD FACS Caliber and statistical analysis was done accordingly.

### Statistical analysis

All data are representative of three independent experiments, unless otherwise mentioned in the figure legends. Statistical analysis was performed using GraphPad Prism 8.0. The difference between the control and experimental groups was analysed by Student's *t*-test. The error bars indicate mean±s.d.

## Supplementary Material

Supplementary information

Reviewer comments
