# Supplementary material for: PCTAIRE1 promotes mitotic progression and resistance against antimitotic and apoptotic signals
Source: J Cell Sci. 2022 Feb 9;135(3):jcs258831. doi: 10.1242/jcs.258831 (PMC8918779; doi:10.1242/jcs.258831)
Supplement: Supplementary information [file joces-135-258831-s1.pdf]

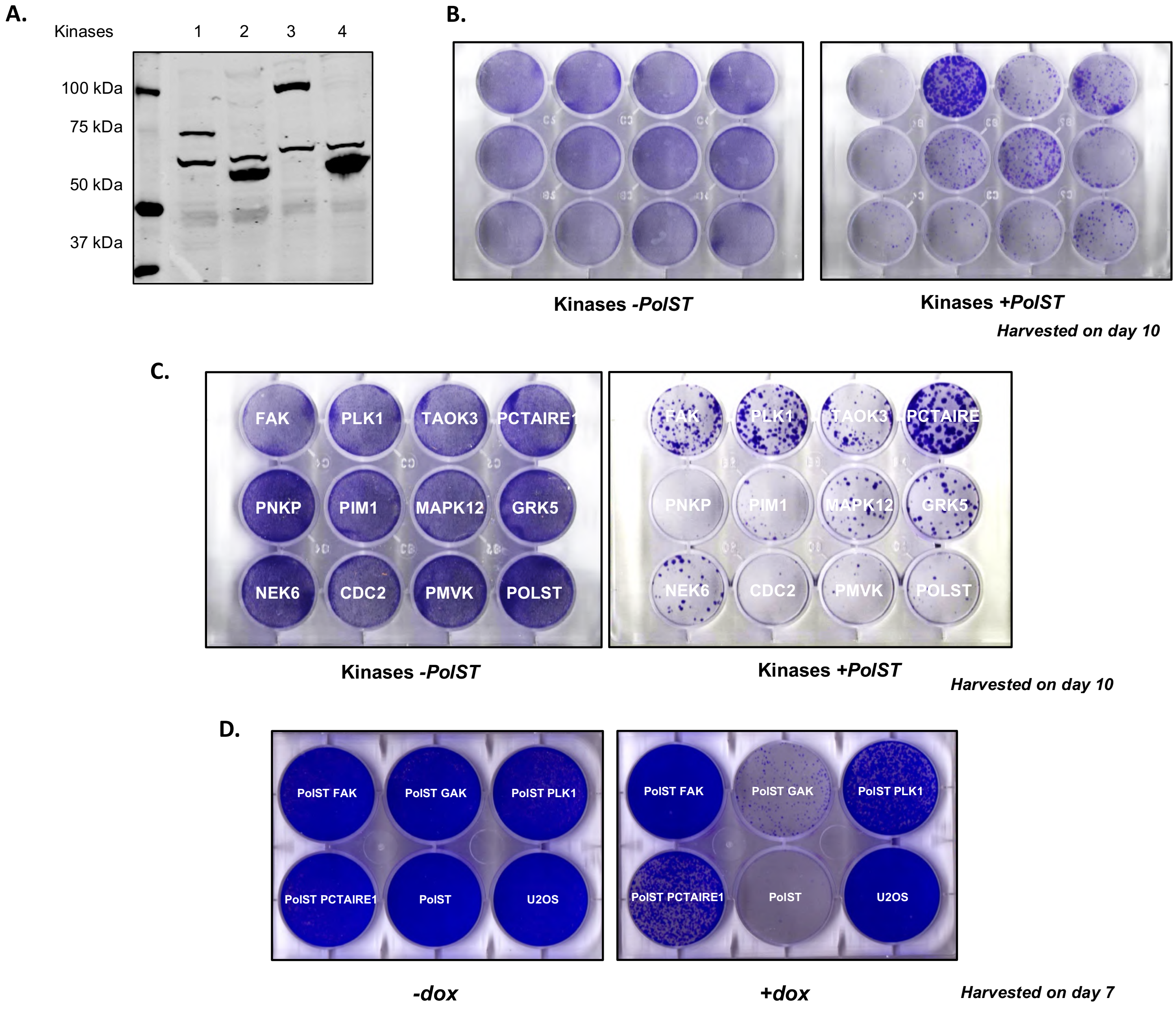

Fig. S1. (A) Western blot showing the expression of some of the candidates of the kinase library in stable cell lines using anti-Flag antibody. (B) Stable cell lines of all the kinases were seeded in batches of 12 each and subjected to primary screening by inducing PolST expression using doxycycline. Control plate was harvested ad stained by crystal violet on the next day. Cells in the experimental plate were monitored each day and replenished with fresh media and doxycycline every alternate day. Cells were harvested on day 10 after seeding and the wells with substantial cell survival in comparison to the control PolST well were identified and shortlisted. (C) The shortlisted candidates from primary screening were seeded in 12 well plates along with a couple of negative candidates and PolST control, and subjected to additional rounds of screening in presence of PolST expression. (D) Some of the positive candidates were cloned as non-myristoylated versions in different vectors and used for making stable cell lines, which were subjected to another round of screening for 7 days. With the exception of Cyclin G associated kinase (GAK), all the other positive candidates reproducibly promoted cell survival in all rounds of screenings.

Journal of Cell Science • Supplementary information

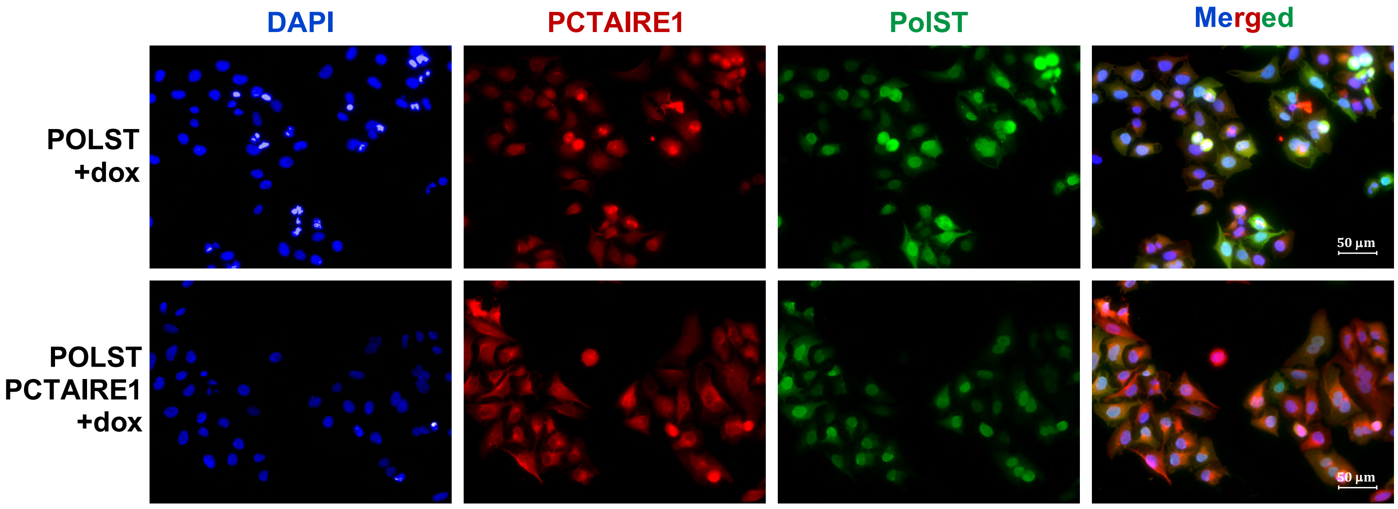

Fig. S2. Immunofluorescence results showing the expression of both PCTAIRE1 and PolST in the cells. The PCTAIRE1 overexpressing U2OS cells showed much better cellular morphology than the control cells. PCTAIRE1 expression was detected by anti-PCTAIRE1 antibody. Cells undergoing mitosis were identified by condensed (light blue) nuclei as seen in DAPI staining.

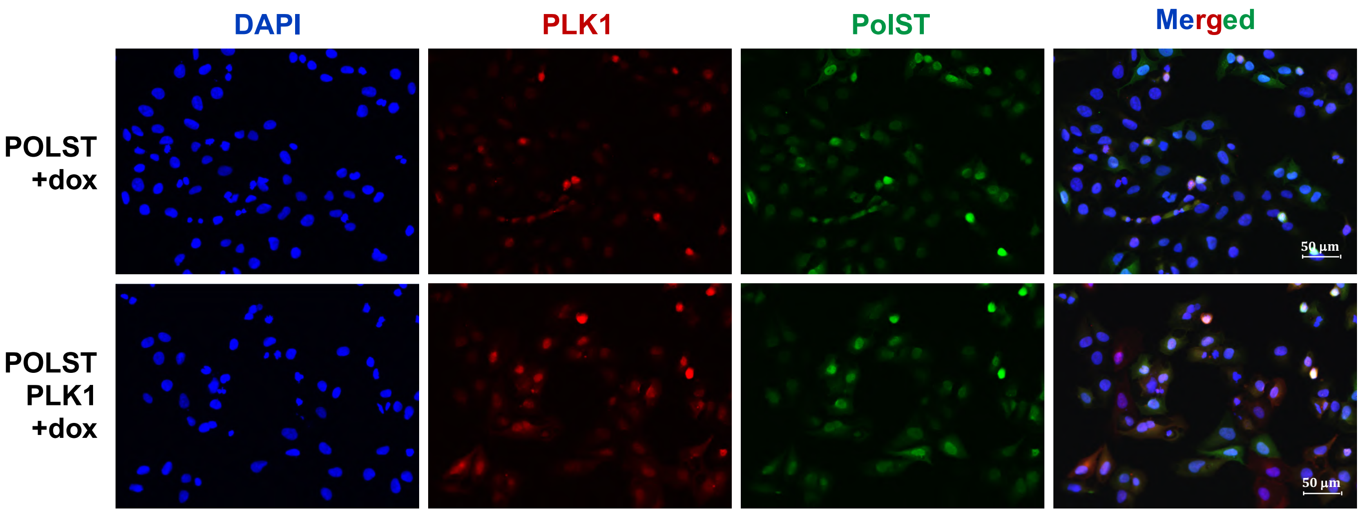

Fig. S3. Immunofluorescence results showing expression of both PLK1 and PolST in U2OS PLK1-PolST stable cell lines. The percentage of cells with better cellular morphology was higher in presence of PLK1 overexpression. DAPI was used for nuclear staining and anti-PLK1 and anti-FLAG antibodies were used to detect PLK1 and PolST expressions, respectively.

Journal of Cell Science • Supplementary information

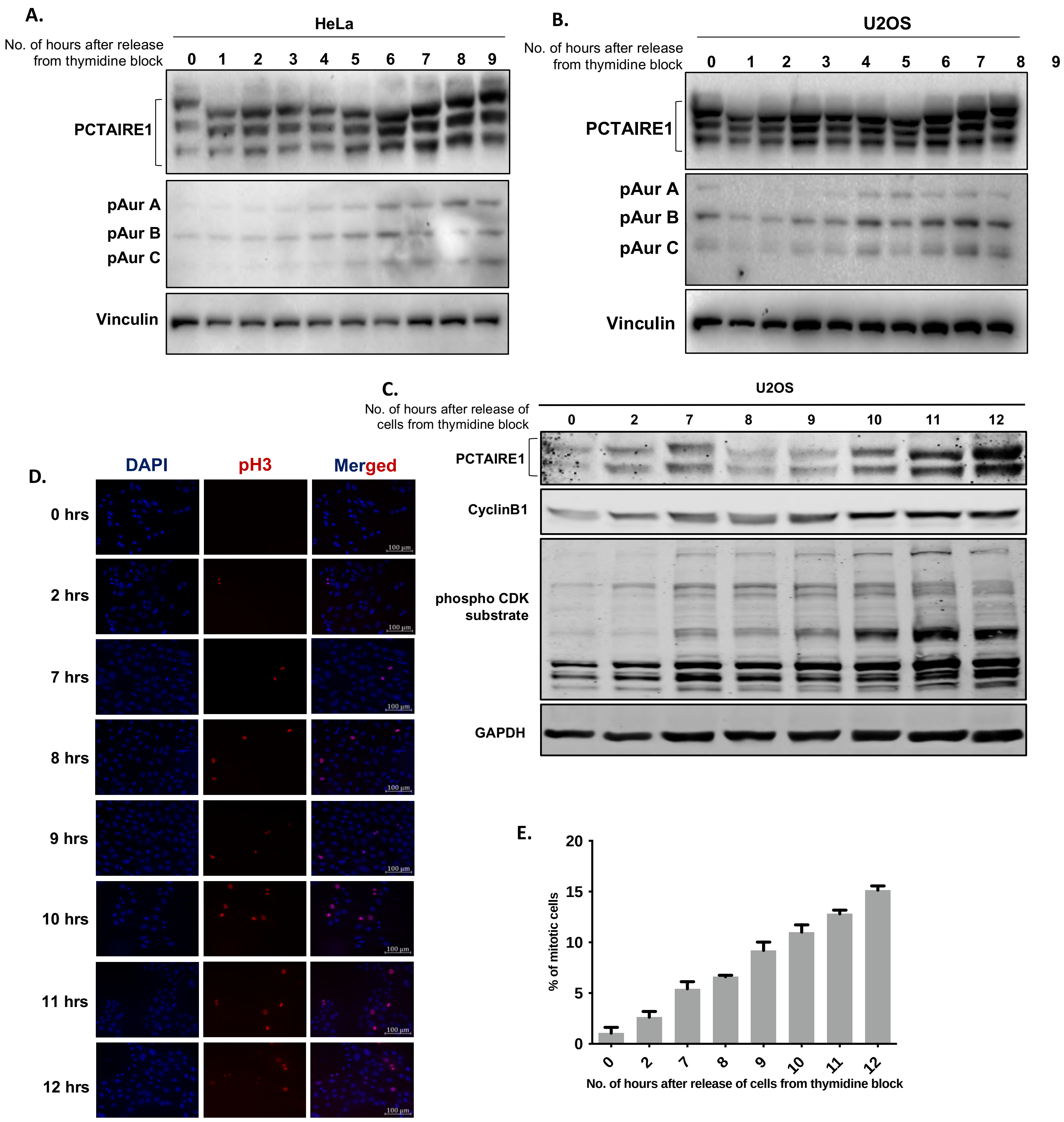

Fig. S4. (A,B) Western blot showing increased PCTAIRE1 protein amounts as well as phosphorylation levels, as indicated by reduced band mobilities in U2OS and HeLa cells respectively, as the cells progressed towards mitosis after release from thymidine block. Mitotic stage was apparent at about 6–7 hrs after thymidine release as was confirmed by enhanced phospho-Aurora kinase signals. (C) Western blot showing increased PCTAIRE1 protein amounts in U2OS cells as the cells progressed towards mitosis after release from thymidine block, as reflected by increased cyclin B1 and phospho-CDK substrate levels. (D) Mitotic index for cells in figure C was also monitored by immunofluorescence analysis of phospho-Histone 3 along with DAPI staining. (E) Graphical representation of the mitotic index of cells as shown in figure D.

Journal of Cell Science • Supplementary information

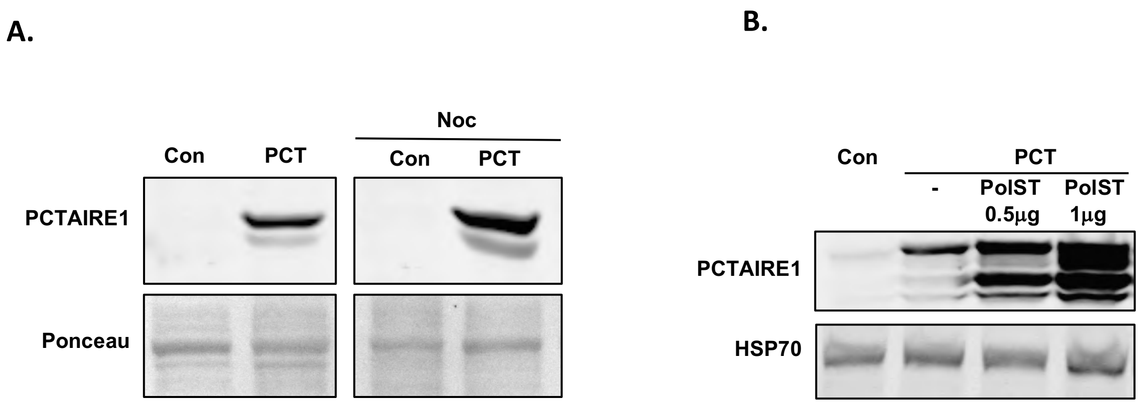

Fig. S5. (A) PCTAIRE1 levels increased in transiently transfected 293T cells in presence of nocodazole (50 ng/ml for 24 hours). 1 μg of PCTAIRE1 was transfected into the cells. Ponceau staining was used to indicate equal loading of protein amounts. (B) Western blot showing the stabilization of PCTAIRE1 protein levels in presence of different amounts of PolST after transient transfections of 0.5 μg and 1.0 μg of PolST-HA-FLAG with 1.0 μg PCTAIRE1-FLAG in 293T cells. PCTAIRE1 expression was detected by using anti-FLAG antibody and PolST expression was detected by using anti-HA antibody. HSP70 was used as a loading control.

Journal of Cell Science • Supplementary information

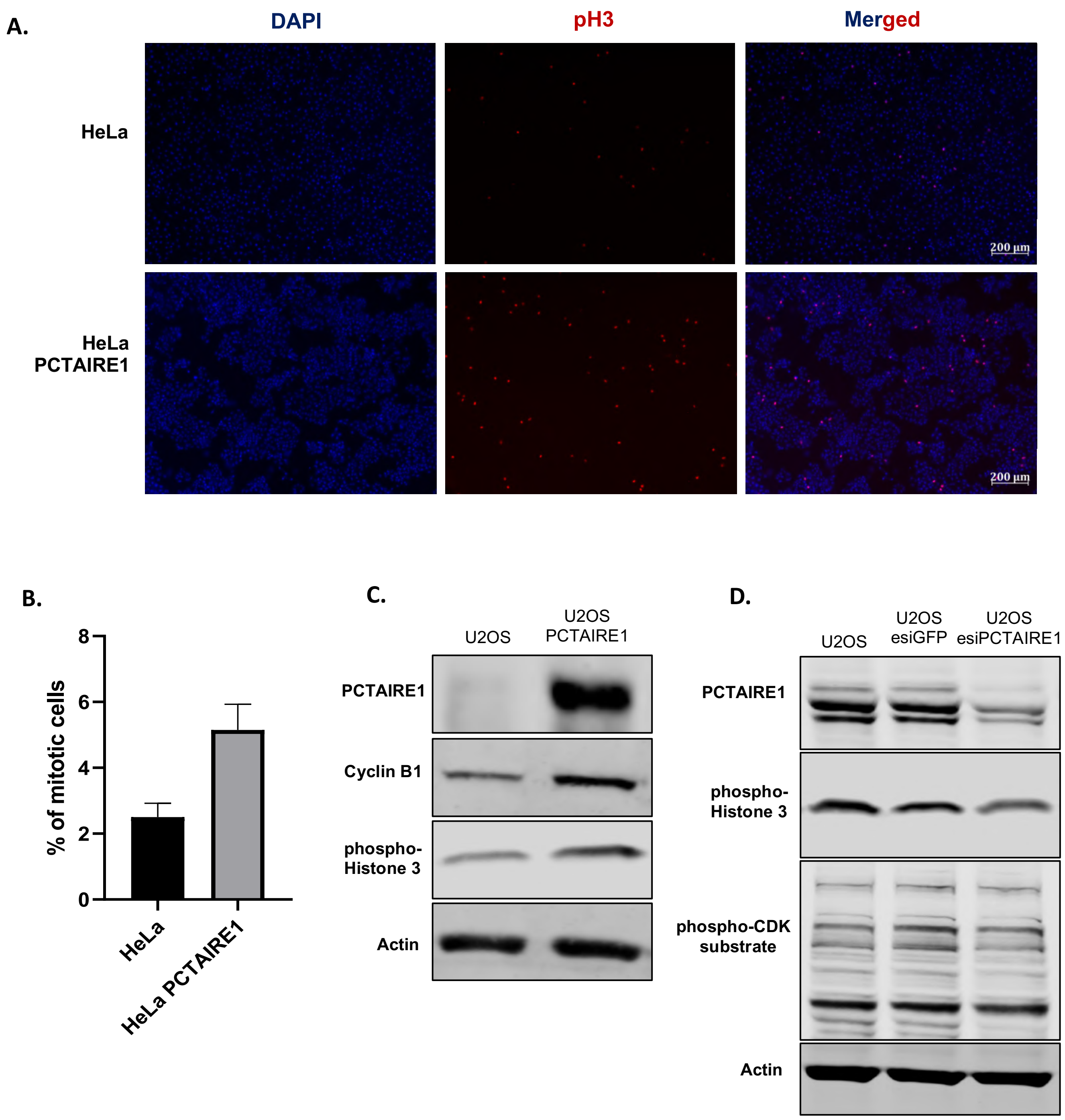

Fig. S6. (A,B) PCTAIRE1 overexpression in HeLa cells increased the percentage of mitotic cells as observed by phospho-Histone 3 staining using anti-phospho-Histone 3 antibody (5X magnification). (C) Western blot showing the overexpression of PCTAIRE1in U2OS cells. Cyclin B1 and phospho-Histone 3 levels were increased in PCTAIRE1 overexpression lane. (D) Western blot showing the knockdown of PCTAIRE1 in cell lysates harvested after 72 hours of esiRNA PCTAIRE1 transfection in U2OS cells. phospho-Histone 3 and phospho-CDK substrate levels were decreased in PCTAIRE1 knockdown lane.

Journal of Cell Science • Supplementary information

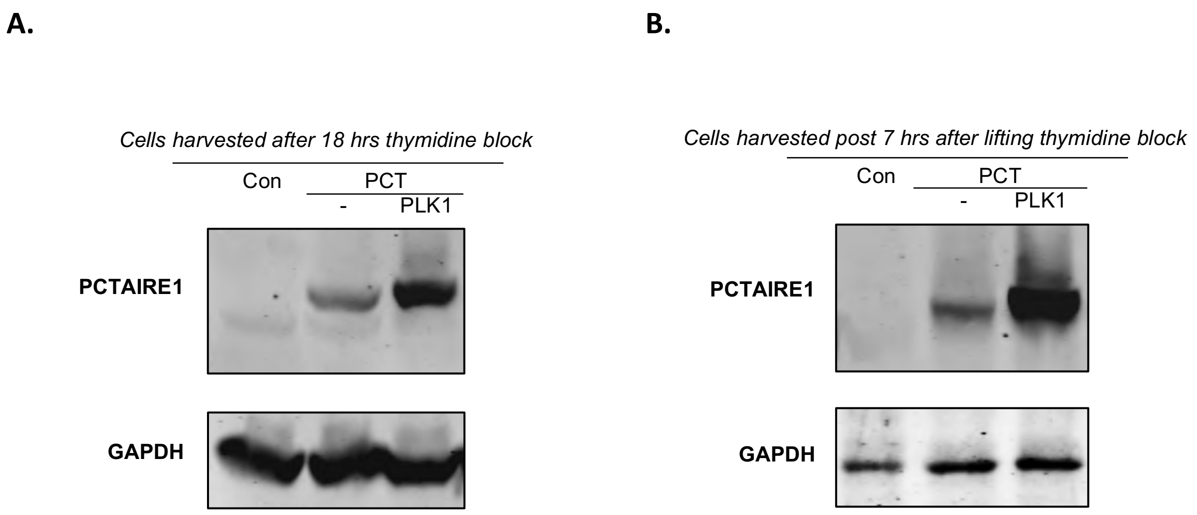

Fig. S7. (A) In cells that were collected immediately after thymidine block for 18 hours (interphase cells), PCTAIRE1 levels were seen to be stabilized when 293T cells were co-transfected with PCTAIRE1-FLAG (1μg) and PLK1-myc-tag (1μg). (B) Following co-transfections of PCTAIRE1-FLAG (1μg) with PLK1-myc-tag (1μg) in 293Ts, cells were blocked by thymidine for 18 hours. After release of cells from thymidine block for 7 hours, PCTAIRE1 was seen to get stabilized by both PLK1.

Journal of Cell Science • Supplementary information

A.

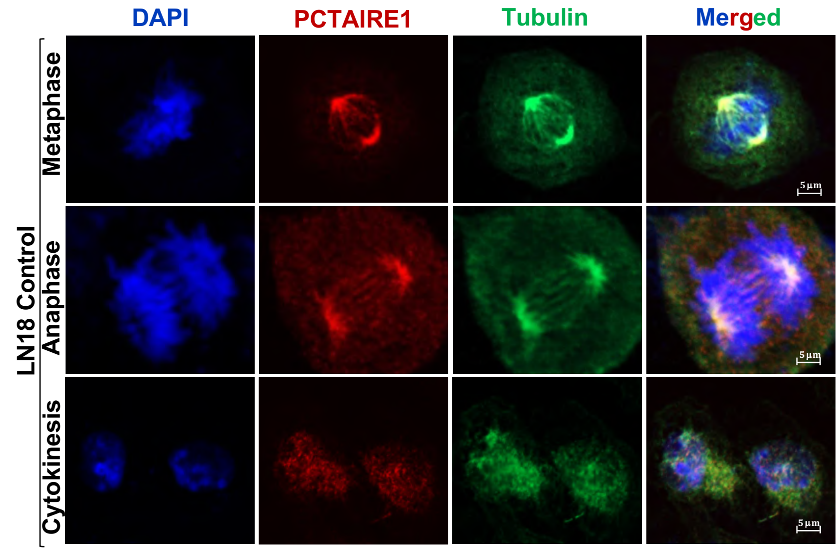

B.

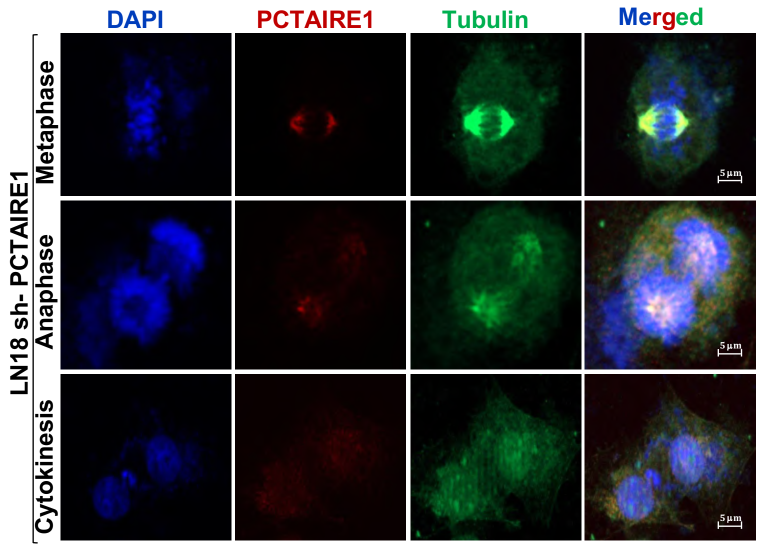

Fig. S8. (A,B) Tubulin and PCTAIRE1 staining in control and shPCTAIRE1 LN18 cells, respectively. Aberrant mitosis can be seen in LN18 cells after PCTAIRE1 knockdown, after induction for 72 hours.

Journal of Cell Science • Supplementary information

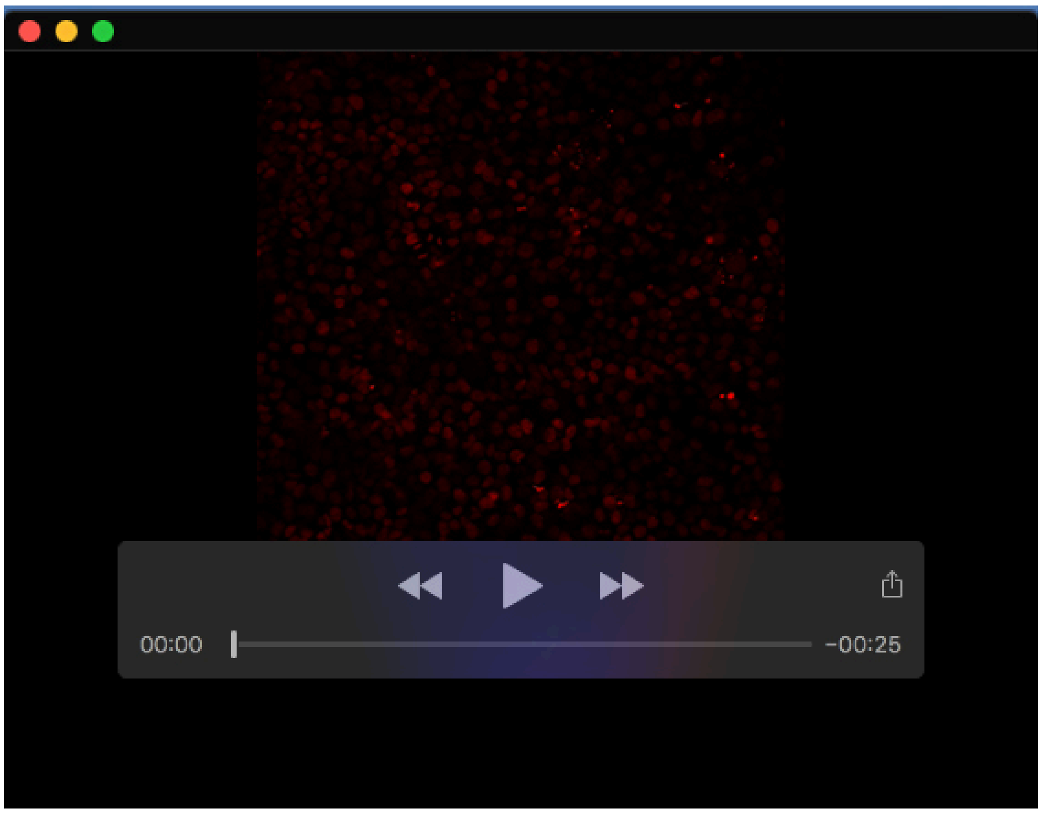

Movie 1: PolST-dox cells. Cells can be observed undergoing normal mitotic divisions throughout the movie.

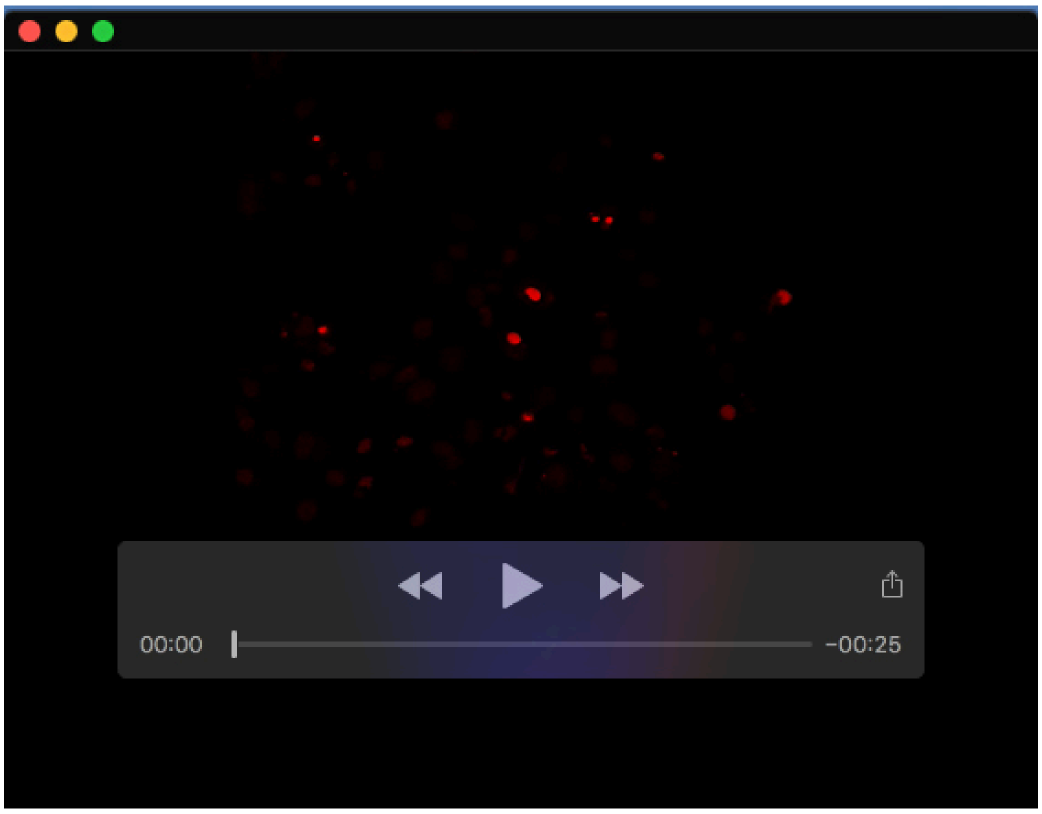

Movie 2: PolST+dox cells expressing PolST for 72 hours and monitored during the last 16 hours of this period. Most of the cells exhibit phenomenal mitotic arrest and are delayed in a prometaphase-like stage, whereas many others undergo apoptosis.

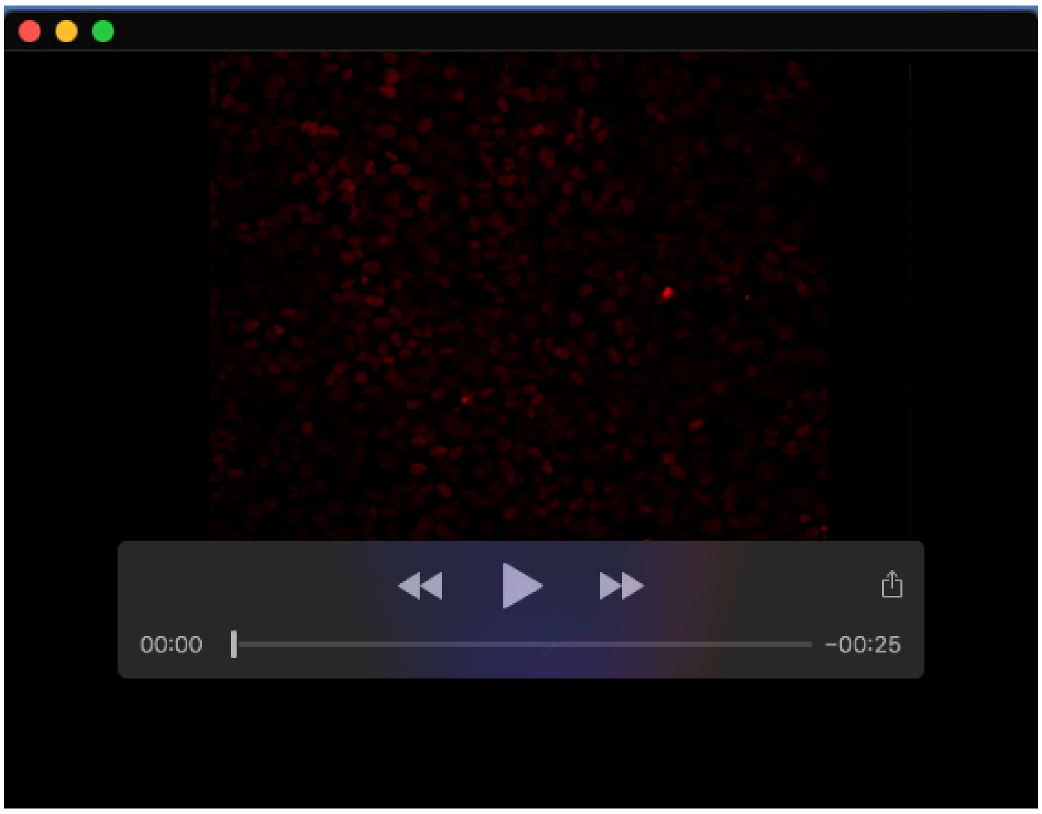

Movie 3: PolST PCTAIRE1-dox cells. Cells can be observed undergoing normal mitotic divisions throughout the movie.

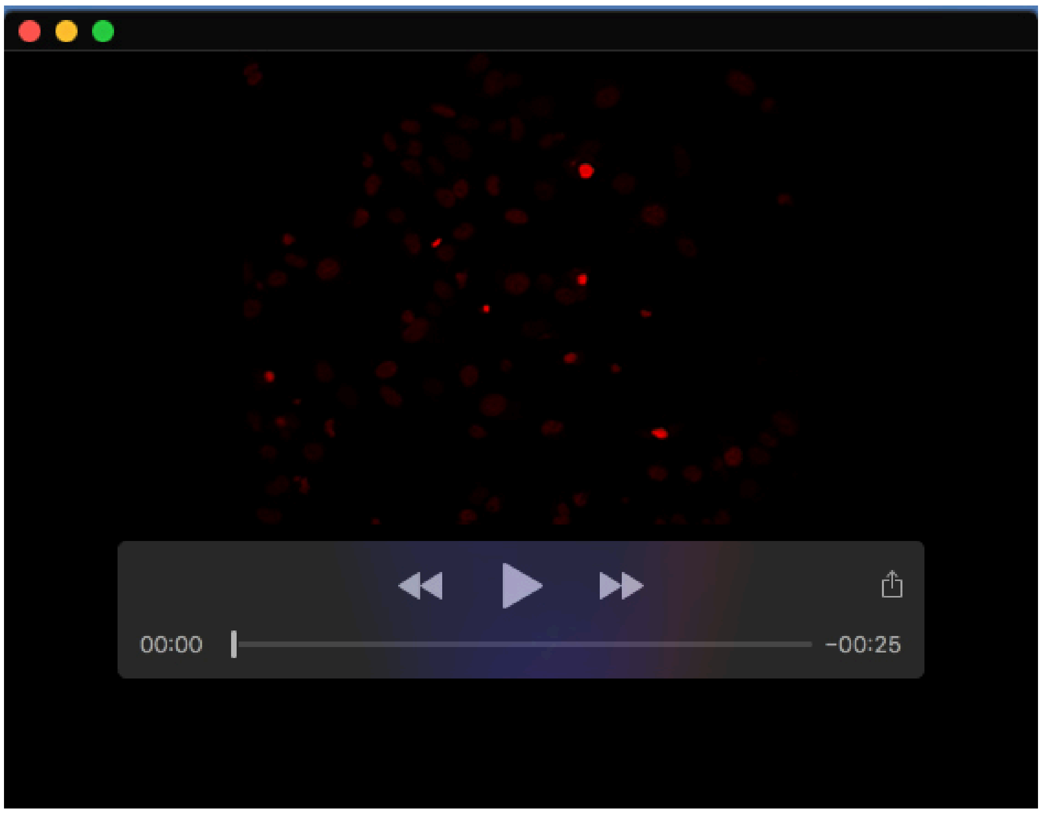

Movie 4: PolST PCTAIRE1+dox cells expressing PolST for 72 hours and monitored during the last 16 hours of this period. Many cells can be seen to divide successfully; however, the division is delayed for some of the cells.

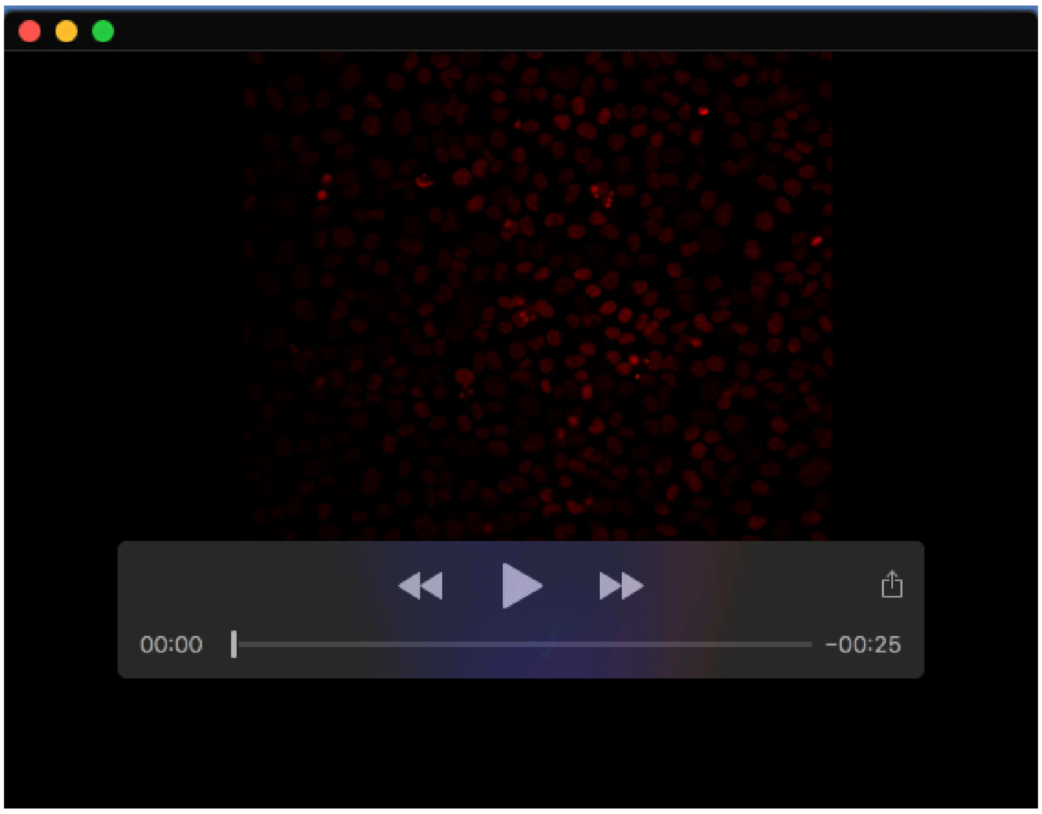

Movie 5: PolST-dox cells. Cells can be observed undergoing normal mitotic divisions throughout the movie.

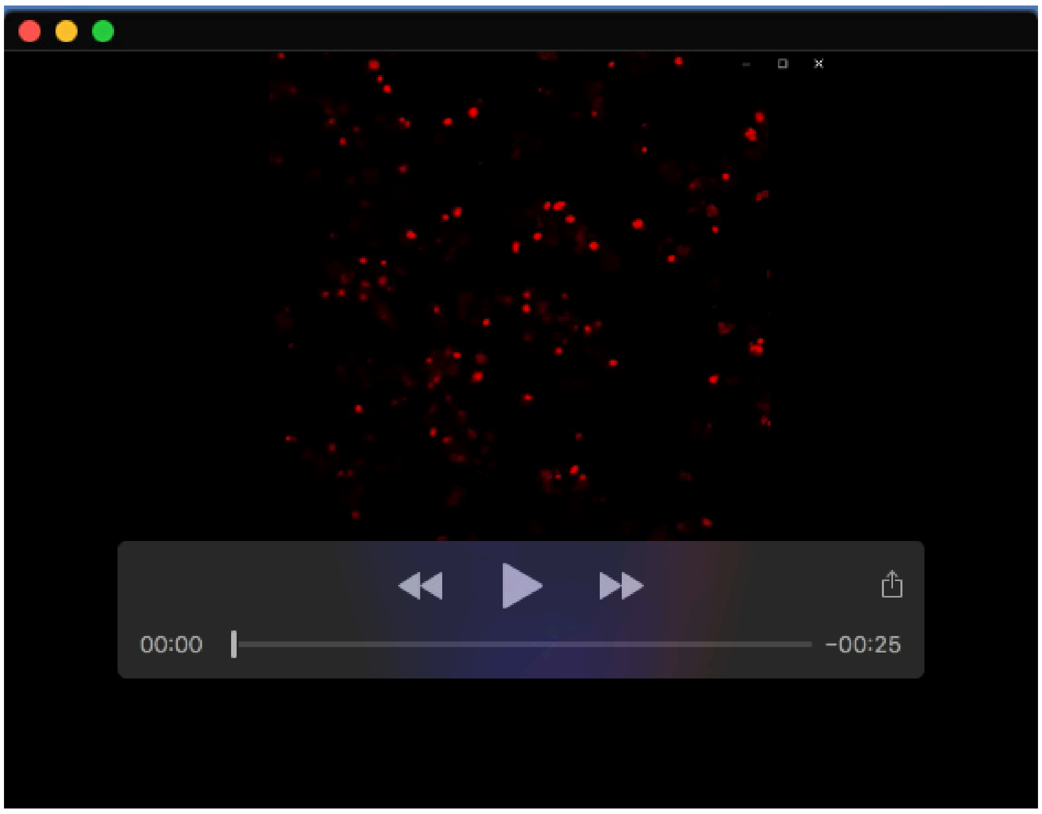

Movie 6: PolST+dox cells expressing PolST for 48 hours and monitored during the last 16 hours of this period. Most of the cells exhibit phenomenal mitotic arrest and are delayed in a prometaphase-like stage, whereas many others undergo apoptosis.

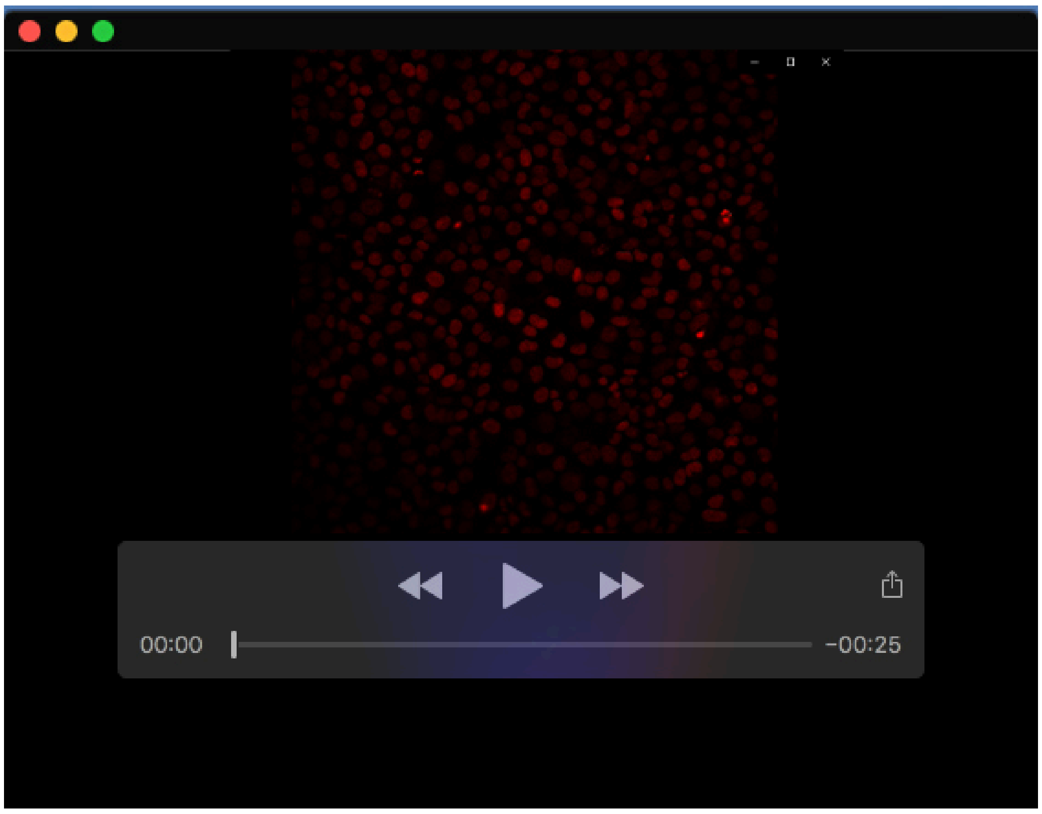

Movie 7: PolST PLK1-dox cells. Cells can be observed undergoing normal mitotic divisions throughout the movie.

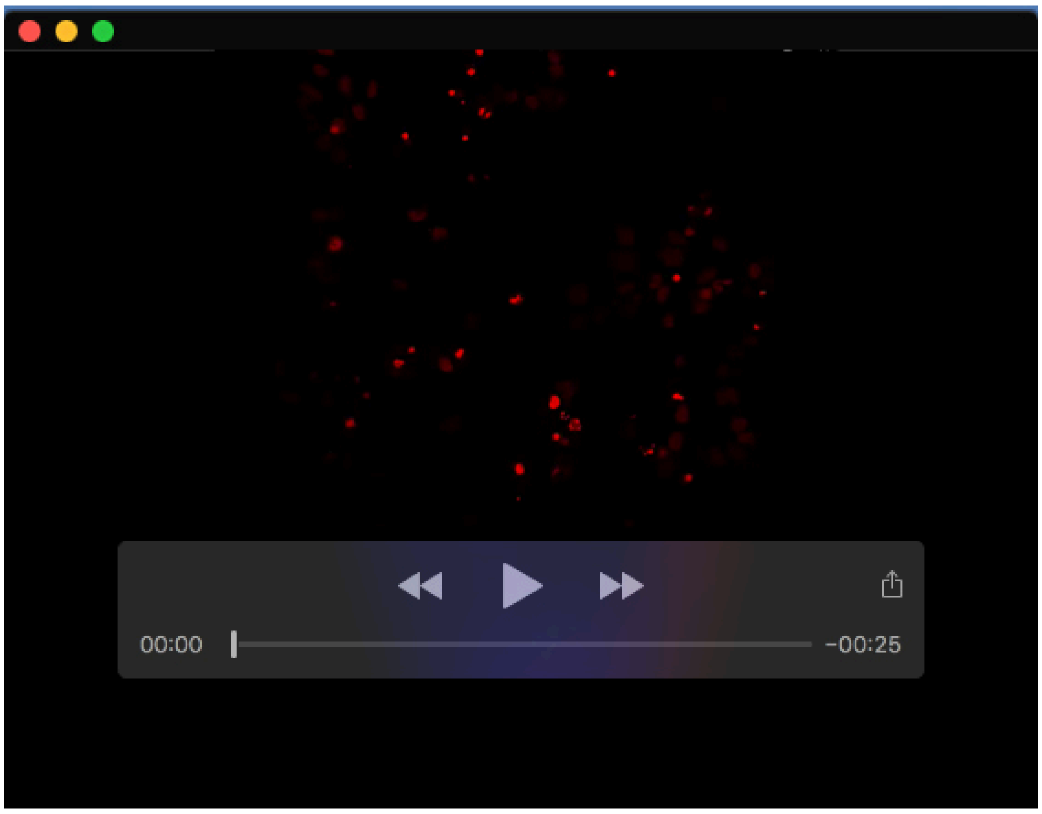

Movie 8: PolST PLK1+dox cells expressing PolST for 48 hours and monitored during the last 16 hours of this period. Many cells can be seen to divide successfully; however, the division is delayed for some of the cells.

Journal of Cell Science • Supplementary information
