# Supplementary material for: PCTAIRE1 promotes mitotic progression and resistance against antimitotic and apoptotic signals
Source: J Cell Sci. 2022 Feb 9;135(3):jcs258831. doi: 10.1242/jcs.258831 (PMC8918779; doi:10.1242/jcs.258831)
Supplement: Reviewer comments [file joces-135-258831_review_history.pdf]

PCTAIRE1 promotes mitotic progression and resistance against antimitotic and apoptotic signals

Syed Qaaifah Gillani, Irfana Reshi, Nusrat Nabi, Misbah Un Nisa, Zarka Sarwar, Sameer Bhat, Thomas M. Roberts, Jonathan M. G. Higgins and Shaida Andrabi
DOI: 10.1242/jcs.258831

Editor: David Glover

Review timeline

Original submission:	23 April 2021
Editorial decision:	14 June 2021
First revision received:	6 October 2021
Editorial decision:	6 December 2021
Second revision received:	14 December 2021
Accepted:	29 December 2021

Original submission

First decision letter

MS ID#: JOCES/2021/258831

MS TITLE: PCTAIRE1 promotes mitotic progression and resistance against antimitotic and apoptotic signals

AUTHORS: Shaida Andrabi, Syed Qaaifah Gillani, Irfana Reshi, Misbah Un Nisa, Zarka Sarwar, Sameer Ahmed Bhat, Nusrat Nabi, Thomas M Roberts, and Jonathan M.G Higgins
ARTICLE TYPE: Research Article

We have now reached a decision on the above manuscript.

To see the reviewers' reports and a copy of this decision letter, please go to: https://submit-jcs.biologists.org and click on the 'Manuscripts with Decisions' queue in the Author Area. (Corresponding author only has access to reviews.)

As you will see, the reviewers raise a number of substantial criticisms that prevent me from accepting the paper at this stage. They suggest, however, that a revised version might prove acceptable, if you can address their concerns. If you think that you can deal satisfactorily with the criticisms on revision, I would be pleased to see a revised manuscript. We would then return it to the reviewers.

We are aware that you may be experiencing disruption to the normal running of your lab that makes experimental revisions challenging. If it would be helpful, we encourage you to contact us to discuss your revision in greater detail. Please send us a point-by-point response indicating where you are able to address concerns raised (either experimentally or by changes to the text) and where you will not be able to do so within the normal timeframe of a revision. We will then provide further guidance. Please also note that we are happy to extend revision timeframes as necessary.

Please ensure that you clearly highlight all changes made in the revised manuscript. Please avoid using 'Tracked changes' in Word files as these are lost in PDF conversion.

© 2022. Published by The Company of Biologists under the terms of the Creative Commons Attribution License (https://creativecommons.org/licenses/by/4.0/).

I should be grateful if you would also provide a point-by-point response detailing how you have dealt with the points raised by the reviewers in the 'Response to Reviewers' box. Please attend to all of the reviewers' comments. If you do not agree with any of their criticisms or suggestions please explain clearly why this is so.

Reviewer 1

Advance summary and potential significance to field

The authors reported the role for PCTAIRE1 (CDK16) in mitosis and anti-apoptosis.
They have identified PCTAIRE1 through kinome library screening approach. They have clarified the localization of PCTAIRE1, and interactors of PCTAIRE1. Further, they have shown the results of knockdown and overexpression of PCTAIRE1. The significance of this study is the critical role of PCTAIRE1 role in mitosis.

Comments for the author

The authors have submitted quite a fascinating manuscript. This is well written and illustrated paper. The paper is novel in its approach (kinome screening approach).
I have some minor comments.
Figure 5 a and b: The authors concluded that "PCTAIRE1 levels peaked during mitosis." To support this conclusion, the authors should perform cell cycle analysis using Flowcytometry.
Figure 5: The authors mentioned that "the phosphorylation levels of PCTAIRE1 are indicated by its reduced band mobilities". Please cite the reference to support this conclusion.

Reviewer 2

Advance summary and potential significance to field

In this manuscript, Gillani and co-workers describe the identification of protein kinases that reduce sensitivity of cells to overexpression of the polyoma virus small T antigen (PolST). The initial part of the manuscript describes a screen using a library of 200 serine-threonine protein kinases in cells with a doxycycline inducible PolST transgene. Induction of PolST results in apoptotic cell death and prolonged arrest in mitosis. A number of kinases increased cell survival, and of these CDK16 and PLK1 were examined further in the manuscript. The authors show that CDK16 localises to the mitotic spindle and may interact with both PLK1 and PP2A. CDK16 depletion results in early mitotic defects, suggesting a role in spindle formation, consistent with this localisation. They then discuss a link to the mitotic spindle and cytokinesis factor PRC1, which is regulated by PP2A and PLK1. The finding are interesting but could be developed further with some simple additional experiments to test this idea. It would be helpful to more clearly separate what CDK16 is normally doing, and why its overexpression “rescues” or suppresses cell death when PolST is overexpressed. How much is what is seen in PolST expressing cells is explained by alterations in PP2A regulation of PRC1?

Comments for the author

Specific comments:
The initial screen data are convincing in terms of the images of the plates. I would have liked to see some quantitative measure to show how reproducible the effects were. This applies to a number of figures in the manuscript where plates of cells are shown.
PolST is thought to cause mitotic delays and trigger apoptosis by uncoupling PP2A catalytic subunits from their normal cellular partners. This may divert PP2A to different substrate proteins, leaving other protein hyperphosphorylated and thus explaining the mitotic delay. Why should overexpression of a kinase, and increasing phosphorylation further, suppress the effect of PolST overexpression? Given the previous published work, it may be worthwhile looking at PRC1 phosphorylation to find out if this is altered when PolST is overexpressed and what effects CDK16 has. Commercial antibodies to the CDK-sites exist and could be used for this purpose.
Why is a library of myristoylated protein kinases used for the initial screen? This is unclear and the results obtained with non-myristoylated kinases appear similar. This isn’t entirely expected, since

© 2022. Published by The Company of Biologists under the terms of the Creative Commons Attribution License (https://creativecommons.org/licenses/by/4.0/).

for some kinases one might have expected that localisation plays an important role. This is known to be the case for PLK1 for example.
The authors test two proteins for interaction with CDK16 – PP2A and PLK1. Both interact, which is somewhat unsatisfactory and additional specificity controls would be helpful.
Can the authors clarify if CDK16 has been reported in any of the published PLK1 interactomes, and how PLK1 binds to CDK16? Is this phosphorylation dependent and via the polo-box domain? This could be tested fairly simply using known mutants of PLK1. A related question is where on CDK16 does PLK1 bind – is there a defined region or site? Knowing this would allow some experiments to test if the interaction is required to suppress the PolST phenotype or for normal cell divisions.
PRC1 is known to bind to PLK1 in anaphase cells. Is this altered in cells overexpressed PolST? How does this change, if at all, when CDK16 or PLK1 are overexpressed as in the screen conditions?
Depletion of CDK16 does not prevent PLK1 localisation in anaphase, so is the role for CDK16 only seen if PolST is present?
CDK16-depleted U2OS cells seem to display early mitotic phenotypes. However, the time of the experiment is not clearly indicated so these may be secondary defects. Have the authors performed a careful time course analysis to see which changes occur first?
What percentage of cells show the altered midbody position phenotype in Figure 7a?

<u>Reviewer 3</u>

Advance summary and potential significance to field

Previous work showed the expression of PolST leads to mitotic arrest and death in U2OS cells. The current paper presents a screen for protein kinases that lessen the toxicity of PolST, plus a detailed characterization of two such kinases: PCTAIRE1 and PLK1, with particular emphasis on the less-well-studied kinase PCTAIRE1.
The main points are: PCTAIRE1 overexpression leads to prolonged mitosis and resistance to pacitaxel and nocodazole; PCTAIRE1 knockdown sensitizes cells a bit to paclitaxel and nocodazole; the endogenous PCTAIRE1 protein increases in abundance after thymidine block and release; endogenous PCTAIRE1 is present in the spindle and midbody during mitosis; and knocking down PCTAIRE1 causes mitotic arrest and apoptosis.
Taken together these findings show that PCTAIRE1 overexpression can lessen the effects of PolST expression and that PCTAIRE1 may play a role in normal mitotic progression.

Comments for the author

The paper is clearly written and well-focused. I have two main criticisms of it, though:
1. Activating PP2A by PolST expression presumably promotes premature mitotic entry, with the cells then trapped in mitosis due to checkpoint activation or to the cells' inability to exit mitosis through a direct effect on APC or PP2A, or to both checkpoint and direct effects. The authors do show that the duration of mitosis is greatly prolonged upon PolST expression, and is normalized when PCTAIRE1 is overexpressed. But we do not know whether this is because PCTAIRE1 turns PolST off, or PCTAIRE1 overexpression inhibits mitotic entry, or PCTAIRE1 overexpression promotes mitotic exit (or all of these). Live cell microscopy should be able to get at this issue. That said I would be willing to forgo this additional information--in general I think that reviewers should not ask for an expansion in the scope of the work, especially in this pandemic year. But I am still left with point 2 below.
2. On many key points, the evidence in the figures and movies is much less convincing than the text would lead you to believe it is. Here are a few examples:
• In Figure 2, the cells stain for PCTAIRE1 (Fig 2c, red) or PLK1 (Fig 3c, red) even when they are not expressing the kinase. These data are supposed to show that the rescued cells are actually overexpressing these kinases, but the background fluorescence is too high to allow that conclusion to be drawn.
• The data on the duration of mitosis (e.g. Fig 2i) looks very convincing, but to judge the duration of mitosis you need to have a reliable gauge of when mitosis begins and when it ends. The authors say they are judging mitotic onset from NEBD. But I cannot see when NEBD takes place in the montages of the live cell imaging shown in Fig 2e and f (especially 2e). Anaphase onset is easy to see (and I presume they are using this rather than the harder-to-gauge timing of cytokinesis as the time of mitotic exit), but without a confident identification of NEBD or some other measure of

© 2022. Published by The Company of Biologists under the terms of the Creative Commons Attribution License (https://creativecommons.org/licenses/by/4.0/).

the timing of mitotic onset, it is hard to be sure about mitotic duration. I can judge mitotic onset better from the movies, although it is chromatin condensation I am gauging, not NEBD. Perhaps if the authors measure the DNA staining fluorescence intensity as a function of time, they will be able to graphically show when chromatin condensation happens from a sudden increase in brightness. Or perhaps higher magnification microscopy would help. Or a different mitotic marker.

• The authors say that PCTAIRE1 levels peak in mitosis. But in Figs 5a and b, it is still increasing (or at least not yet decreasing) in the latest time points. And they say there is a "marked" increase in its phosphorylation.

If there is any increase in phosphorylation beyond what you would expect just from a gradual increase in abundance, it is subtle. And it is not demonstrated that the multiple putative PCTAIRE1 bands represent different levels of phosphorylation. This could and should be checked by treating samples with lambda phosphatase ± vanadate.

• Given the background fluorescence in Fig 2, I am not convinced that the signal in Fig 6 represents PCTAIRE1. Does knocking down PCTAIRE1 make the signal go away?

• Can the PCTAIRE1-esiRNA knockdown phenotype be rescued by expressing PCTAIRE1 from a different species, or by using siRNA made from the 3'-UTR and rescuing with a construct that lacks the 3'-UTR?

If the authors can firm-up these main experimental findings, I could support publication of this paper, but as it is I am intrigued but not convinced.

Minor points:

3. Line 46: "PCTAIRE1, also known as cyclin dependent kinase 16 (CDK16 or PCTK1) (Malumbres et al., 2009), belongs to the poorly-characterized family of cyclin dependent kinases (CDKs) which includes PCTAIRE 1, 2 and 3 (Manning et al., 2002)." Since some members of the CDK family are well-characterized how about change "the poorly-characterized family" to "a poorly-characterized sub-family".
4. Line 96: I think the authors are missing a word--maybe "using"--before the word "PolST".
5. Line 107: What was the rationale behind expressing myristoylated kinases rather than unaltered kinases for the screen? I appreciate that for some kinases, e.g. Akt, forcing the kinase to the membrane is sufficient to activate the kinase, but I would think that only a few of the 200 kinases would be activated this way. Was there some other reason? It would be helpful for the authors to explain this.
6. Line 124: GAK expression appeared to rescue viability in Fig 1c, but it was not considered a positive.

   From the supplement, I think the rescue shown in Fig 1c proved not to be reproducible. If so this should be stated clearly in the main paper.
7. Fig 2i, 3g. These panels are not "dot blots".
8. Fig 4. Here the authors show that PCTAIRE1 overexpression renders cells less sensitive to taxol and nocodazole. Can the authors estimate how overexpressed, on average, PCTAIRE1 is relative to the endogenous protein?
9. Line 632: "were the cells were" should read "where the cells were".

First revision

Author response to reviewers' comments

Response to the queries of reviewers:

Reviewer 1: Advance Summary and Potential Significance to Field:

The authors reported the role for PCTAIRE1 (CDK16) in mitosis and anti-apoptosis. They have identified PCTAIRE1 through kinome library screening approach. They have clarified the localization of PCTAIRE1, and interactors of PCTAIRE1. Further, they have shown the results of knockdown and overexpression of PCTAIRE1. The significance of this study is the critical role of PCTAIRE1 role in mitosis.

© 2022. Published by The Company of Biologists under the terms of the Creative Commons Attribution License (https://creativecommons.org/licenses/by/4.0/).

Reviewer 1 Comments for the Author:
The authors have submitted quite a fascinating manuscript. This is well written and illustrated paper. The paper is novel in its approach (kinome screening approach).

I have some minor comments.

# Figure 5 a and b: The authors concluded that "PCTAIRE1 levels peaked during mitosis." To support this conclusion, the authors should perform cell cycle analysis using Flow cytometry.

Response: We thank the reviewer for this worthy advice. As the flow cytometer of our institute is currently not functional, and we could not get the concerned engineers to fix it due to COVID-19 pandemic, we addressed this concern of the reviewer by using an alternate approach. We repeated the experiment and evaluated the mitotic index using phospho-Histone 3 staining followed by immunofluorescence analysis of the synchronized cells, and parallelly detected PCTAIRE1 protein levels in the cells. We have included this result in the revised manuscript (Supplementary Figure 5).

# Figure 5: The authors mentioned that "the phosphorylation levels of PCTAIRE1 are indicated by its reduced band mobilities". Please cite the reference to support this conclusion.

Response: Phosphorylation is known to lead to reduced mobilities of several proteins, and is particularly commonly found to occur to several mitotic proteins especially during the M-phase (e,g Cdc25, Cdc27, Myt1, Gwl/MastL, Yap1 etc) . Following are some references highlighting this property of such proteins.

- DOI: 10.1158/0008-5472.CAN-13-2049
- DOI: 10.1016/j.molcel.2016.10.034
- DOI: 10.1073/pnas.0914191107
- DOI: 10.1038/emboj.2009.228
- DOI: 10.1083/jcb.201307160
- DOI: 10.1091/mbc.e09-07-0643
- DOI: 10.1074/jbc.C300126200
- DOI: 10.1091/mbc.E10-07-0599
- DOI: 10.1093/emboj/cdf567

In addition, we also have several examples in which expression of small T antigen leads to altered mobilities of some proteins (both increased and reduced mobilities) as can be seen in Western blotting e.g. FOXO1/3, YAP1, lipin-1 etc (DOI: 10.1128/JVI.05034-11; DOI: 10.1128/JVI.01399-14, and unpublished data). In the current manuscript under consideration, we saw similar band shifts (reduced mobilities) for PCTAIRE1 in Fig. 5A,B. The band shifts were more pronounced in the supplementary Figure S4A,B.

Reviewer 2 Advance Summary and Potential Significance to Field:
In this manuscript, Gillani and co-workers describe the identification of protein kinases that reduce sensitivity of cells to overexpression of the polyoma virus small T antigen (PolST). The initial part of the manuscript describes a screen using a library of 200 serine-threonine protein kinases in cells with a doxycycline inducible PolST transgene. Induction of PolST results in apoptotic cell death and prolonged arrest in mitosis. A number of kinases increased cell survival, and of these CDK16 and PLK1 were examined further in the manuscript. The authors show that CDK16 localises to the mitotic spindle and may interact with both PLK1 and PP2A. CDK16 depletion results in early mitotic defects, suggesting a role in spindle formation, consistent with this localisation. They then discuss a link to the mitotic spindle and cytokinesis factor PRC1, which is regulated by PP2A and PLK1. The findings are interesting but could be developed further with some simple additional experiments to test this idea. It would be helpful to more clearly separate what CDK16 is normally doing, and why its overexpression “rescues” or suppresses cell death when PolST is overexpressed. How much is what is seen in PolST expressing cells is explained by alterations in PP2A regulation of PRC1?

© 2022. Published by The Company of Biologists under the terms of the Creative Commons Attribution License (https://creativecommons.org/licenses/by/4.0/).

Reviewer 2 Comments for the Author:

Specific comments:
# 1. The initial screen data are convincing in terms of the images of the plates. I would have liked to see some quantitative measure to show how reproducible the effects were. This applies to a number of figures in the manuscript where plates of cells are shown.

Response: We thank the reviewer for raising this query as it gave us a chance to incorporate additional data in the manuscript to validate the findings further. In the revised manuscript, we have included some of the primary and secondary screening results which also show the approach that we used during our screening strategy (Supplementary Figure S1). In addition, we have also included quantification data of the crystal violet staining to support the results (Figure 1D).

# 2. PolST is thought to cause mitotic delays and trigger apoptosis by uncoupling PP2A catalytic subunits from their normal cellular partners. This may divert PP2A to different substrate proteins, leaving other protein hyperphosphorylated and thus explaining the mitotic delay. Why should overexpression of a kinase, and increasing phosphorylation further, suppress the effect of PolST overexpression? Given the previous published work, it may be worthwhile looking at PRC1 phosphorylation to find out if this is altered when PolST is overexpressed and what effects CDK16 has. Commercial antibodies to the CDK-sites exist and could be used for this purpose.

Response: The expression of some viral proteins like SV40 small T and polyoma middle T in mammalian cells predominantly leads to the hyperphosphorylation of cellular proteins, but we have seen that polyoma small T expression leads to both hyper- as well as hypo-phosphorylation of target cellular proteins. For example, polyoma small T expression leads to the dephosphorylation of Akt (DOI: 10.1073/pnas.0706696104; DOI: 10.1038/onc.2014.192), lipin-1/lipin-2 (at numerous sites; DOI: 10.1128/JVI.05034-11 and manuscript submitted to JCS) and Yap1 (DOI: 10.1128/JVI.01399-14). However, in the same experiments, it leads to the increased phosphorylation of many other proteins as well (e.g. pP38, pJNK, pGSK3, pPCTAIRE1, pPLK1, pAurK, pCDK1 etc). All these changes are brought about by the interaction of PolST with PP2A. We interpret that the impact of PolST on the phosphorylation of substrates is not universal, but is context-dependent. PolST may interact with a specific combination of PP2A subunits, affect its holoenzyme localization and hence its specificity towards different substrates. So, it is not necessarily true that PolST would lead to further phosphorylation of all the cellular substrates (as is the case for SV40 ST and Polyoma middle T). For example, one possibility is that overexpression of PCTAIRE1 causes it to interact with the appropriate PP2A subunits and displace/titrate out PolST from the PP2A complex (PP2A A-C-PolST), thereby facilitating the formation of a PP2A-PCTAIRE1 complex which may be important for mitotic exit. Alternatively, PCTAIRE1 overexpression could restore phosphorylation of a substrate that is inappropriately dephosphorylated because of the ability of PolST to change the substrate specificity of PP2A. We have already mentioned these possibilities in the “Discussion” section of the manuscript. It is our top priority to find out the details of this mechanism, which will be part of our future communications.

Again, as mentioned in our discussion section, we believe that PRC1 may be an important component of this complex that might facilitate the successful mitotic exit at cytokinesis. However, we raise this only as a discussion point and do not make any strong conclusion about the role of PCTAIRE1 in regulating PRC1. In addition, , because of the prolonged pandemic in India which has taken a serious turn, we have mostly been under lockdown and our university has just started to resume work. It has not been possible to purchase various reagents, including antibodies, from abroad given that flights from many countries to India have been predominantly suspended. We anticipate that it will take several months before we could procure these reagents and carry out such experiments. This part is definitely included in our planned work that we want to carry out on priority, but we believe that it is not necessary to support the conclusions that we make in this manuscript.

© 2022. Published by The Company of Biologists under the terms of the Creative Commons Attribution License (https://creativecommons.org/licenses/by/4.0/).

# 3. Why is a library of myristoylated protein kinases used for the initial screen? This is unclear and the results obtained with non-myristoylated kinases appear similar. This isn't entirely expected, since for some kinases one might have expected that localisation plays an important role. This is known to be the case for PLK1 for example.

Response: This is a valid concern raised by the reviewer. When the work was originally started, the only available kinase library was at Addgene, and this was the myristoylated kinase library that we used for this screen. This library had been successfully used for the identification of IKKB¤ as a breast cancer oncogene (DOI: 10.1016/j.cell.2007.03.052). Myristoylation is often used to constitutively activate kinases. However, the mis- localization of the kinases was always a concern and we carried out additional screenings rigorously to rule out any potential artefacts that might arise because of the myristoylation. For that reason, we also compared the myristoylated vs non-myristoylated kinases for the shortlisted candidates in their rescue against PolST phenotype and have seen that cell survival is more prominent in myristoylated kinases than in their non-myrisoylated counterparts. This is explainable for PCTAIRE1 as well as PLK1, as both of them get activated by myristoylation. In fact, PCTAIRE1 localizes to the membranes with the help of its binding partner, Cyclin Y (CCNY) and/or cyclin Y like (CCNYL), and membrane localization is known to activate PCTAIRE1 by several folds (DOI: 10.1016/j.cellsig.2012.06.018). Most of the proteins that we have identified are already known to localize to the membranes, at least at some stages (e.g. PCTAIRE1, PLK1, GRK5, FAK, TAOK3, MAPK12/p38γ). On the other hand, many proteins like PDK1, AKT etc, which are known to localize to the membranes and get activated by myristoylation did not score positive in this screen, thus showing the specificty of our results. We, however, do not rule out the possibility that we may have missed out some candidates because of the potential mislocalization to the plasma membrane. We have consistently found out that non-myristoylated version of PCTAIRE1 is at least 2-3 times less potent in promoting the survival against PolST induced cell death. This clearly shows that myristoylation was very helpful in the identification of such candidates. As of now, Addgene also provides a full kinase library of 500 candidates which in non-myristoylated and has been successfully used for similar screenings (DOI: 10.1038/nature09627). We have not yet been in a position to initiate a much larger version of this type of screen. It may be possible/worthwhile to do that in the future.

# 4. The authors test two proteins for interaction with CDK16-PP2A and PLK1. Both interact, which is somewhat unsatisfactory and additional specificity controls would be helpful.

Response: We are thankful to the reviewer for the worthy advice. In addition to the results that we have observed in our study, PP2A subunits have been found as PCTAIRE1 interactors in proteomic studies as well. (https://thebiogrid.org/111154). However, to address the reviewer's concerns, we have performed the experiment once again with specificity control proteins (DBC1 and SIRT1) to substantiate our data. The results have been incorporated in the revised manuscript (Figure 5E). Neither of these proteins showed interaction with PCTAIRE1 in these experiments.

# 5. Can the authors clarify if CDK16 has been reported in any of the published PLK1 interactomes, and how PLK1 binds to CDK16? Is this phosphorylation dependent and via the polo-box domain? This could be tested fairly simply using known mutants of PLK1. A related question is where on CDK16 does PLK1 bind– is there a defined region or site? Knowing this would allow some experiments to test if the interaction is required to suppress the PolST phenotype or for normal cell divisions.

Response: We are again thankful to the reviewer for this keen observation and for the worthy suggestions.

Many PLK1 high-throughput interactome screens have been carried out, but no interaction between PCTAIRE1 and PLK1 has been reported yet (References: DOI: 10.1038/sj.emboj.7601683; DOI: 10.1073/pnas.0805139105; DOI: 10.1074/mcp.M111.008540; DOI: 10.1074/mcp.M110.004457). It is possible that the interaction between the two proteins is not very strong and hence does not withstand the rigour of such experiments, or the interaction could be happening very transiently at a specific stage in mitotic cells, like during cytokinesis. PCTAIRE1 sequence does have both potential PCTAIRE1 substrate sites and polo-

© 2022. Published by The Company of Biologists under the terms of the Creative Commons Attribution License (https://creativecommons.org/licenses/by/4.0/).

box binding motifs. So, it is quite possible that the two proteins may interact with each other. In addition, since Plk1 interactomes tend to be large, they are very likely to miss a particular protein. Moreover, at least in the Confetti proteome database for HeLa cells (https://proteomics.swmed.edu/confetti/), PCTAIRE1/CDK16 does not appear to be an abundant protein (ranked approx. 5500 out of approx. 7500), thus also reducing the chances of identifying it in such studies.

With regards to the second question, in order to address the reviewers concerns, we obtained constructs for PLK1 mutants (PLK-KD and PLK-PBD) and have accordingly performed immunoprecipitation experiments using these mutants to confirm the interaction of PCTAIRE1 with PLK1. Our results have, however, shown that the interaction is dependent on both the kinase as well as the polo-box domain of PLK1. We have included these results in the revised manuscript (Fig. 6C).

# 6. PRC1 is known to bind to PLK1 in anaphase cells. Is this altered in cells overexpressed PolST? How does this change, if at all, when CDK16 or PLK1 are overexpressed as in the screen conditions? Depletion of CDK16 does not prevent PLK1 localisation in anaphase, so is the role for CDK16 only seen if PolST is present?

Response: We believe this comment is related to comment 2 of reviewer 2, as mentioned above. We feel that the main connecting point between PCTAIRE1, PLK1, and PolST is likely PP2A. Overexpression of PCTAIRE1 or PLK1 may alter PolST interactions with PP2A and facilitate the proper localization of these proteins at the central spindle/midbody during anaphase to cytokinesis. In the “Discussion”, we speculate about a role for PCTAIRE1 in regulating PRC1, but this is not a core conclusion of the paper and we think that exploring this would be expanding beyond the intended scope of the work. Moreover, this would need obtaining PRC1 antibody from abroad and at the moment, due to the pandemic situation, it would take a long time to carry out this experiment.

# 7. CDK16-depleted U2OS cells seem to display early mitotic phenotypes. However, the time of the experiment is not clearly indicated so these may be secondary defects. Have the authors performed a careful time course analysis to see which changes occur first?

Response: The experiments were done at 72 hours after esiRNA transfection. Further, for shPCTAIRE1, the analysis was done at specific time points after induction (48-96 hours). For later time periods (96 hours), however, we observed apoptosis in many cells (results shown in a separate figure not included in the manuscript).

# 8. What percentage of cells show the altered midbody position phenotype in Figure 7a?

Response: Around 55% of cells exhibited altered midbody position phenotype upon PCTAIRE1 knockdown by esiRNA. However, this includes different alignments, and not necessarily the exact alignment as is observed in the cells shown in Fig. 8A in the revised manuscript (e.g., some cells had the midbody that was perpendicular between the daughter cells instead of running parallelly between them, whereas others had the midbody at abnormal angles with respect to each other).

Reviewer 3 Advance Summary and Potential Significance to Field:
Previous work showed the expression of PolST leads to mitotic arrest and death in U2OS cells. The current paper presents a screen for protein kinases that lessen the toxicity of PolST, plus a detailed characterization of two such kinases: PCTAIRE1 and PLK1, with particular emphasis on the less-well-studied kinase PCTAIRE1. The main points are: PCTAIRE1 overexpression leads to prolonged mitosis and resistance to pacitaxel andnocodazole; PCTAIRE1 knockdown sensitizes cells a bit to paclitaxel and nocodazole; the endogenousPCTAIRE1 protein increases in abundance after thymidine block and release; endogenous PCTAIRE1 is present in the spindle and midbody during mitosis; and knocking down PCTAIRE1 causes mitotic arrest and apoptosis. Taken together these findings show that PCTAIRE1 overexpression can lessen the effects of PolST expression, and that PCTAIRE1 may play a role in normal mitotic progression.

© 2022. Published by The Company of Biologists under the terms of the Creative Commons Attribution License (https://creativecommons.org/licenses/by/4.0/).

Reviewer 3 Comments for the Author:
The paper is clearly written and well-focused. I have two main criticisms of it, though:
# 1. Activating PP2A by PolST expression presumably promotes premature mitotic entry, with the cells then trapped in mitosis due to checkpoint activation or to the cells' inability to exit mitosis through a direct effect on APC or PP2A, or to both checkpoint and direct effects. The authors do show that the duration of mitosis is greatly prolonged upon PolST expression, and is normalized when PCTAIRE1 is overexpressed. But we do not know whether this is because PCTAIRE1 turns PolST off, or PCTAIRE1 overexpression inhibits mitotic entry, or PCTAIRE1 overexpression promotes mitotic exit (or all of these). Live cell microscopy should be able to get at this issue. That said I would be willing to forgo this additional information--in general I think that reviewers should not ask for an expansion in the scope of the work, especially in this pandemic year. But I am still left with point 2, below.

Response: We thank the reviewer for this keen observation and for being so considerate with regards to understanding the difficulty to conduct the elaborate experiment amidst the current pandemic.

We believe that PolST may not necessarily be involved only in activating PP2A. This is because a likely possibility is that PolST may inactivate PP2A activity towards some substrates by displacing it from its regulatory subunits, while in other cases PolST binding to PP2A may make it more active towards other substrates (as explained above). Nevertheless, of course the precise mechanism by which PCTAIRE1 rescues the effects of PolST is of significant interest. Live cell imaging of PolST-expressing cells in this work (Figs. 2 and 3), and in our previous work (DOI: 10.1038/onc.2014.192) did not reveal any changes in the rate at which cells entered mitosis, and there does not seem to be a particular reason to presume that PolST promotes mitotic entry. However, it is true that we do not know if PCTAIRE1 acts to normalise the change in PP2A activity caused by PolST, or whether it promotes mitotic exit in some other way. As agreed by the reviewer, answering the question would require a significant expansion of the work, and the current lockdown restrictions in India would make this particularly challenging at this time.

# 2. On many key points, the evidence in the figures and movies is much less convincing than the text would lead you to believe it is. Here are a few examples:

- In Figure 2, the cells stain for PCTAIRE1 (Fig 2c, red) or PLK1 (Fig 3c, red) even when they are not expressing the kinase. These data are supposed to show that the rescued cells are actually overexpressing these kinases, but the background fluorescence is too high to allow that conclusion to be drawn.

Response: We would like to clarify that the antibodies used for these experiments were against PCTAIRE1 and PLK1 (not the tags), respectively, and therefore, they stained endogenous PCTAIRE1 and PLK1 as well.
Nevertheless, the difference in their expression levels can be clearly seen in case of overexpressed PCTAIRE1 and PLK1 cells. So, in the -dox panels, these antibodies have stained the endogenous PCTAIRE1 and PLK1 also (Revised manuscript Supplementary information, Figures S2 and S3).

- The data on the duration of mitosis (e.g. Fig 2i) looks very convincing, but to judge the duration of mitosis you need to have a reliable gauge of when mitosis begins and when it ends. The authors say they are judging mitotic onset from NEBD. But I cannot see when NEBD takes place in the montages of the live cell imaging shown in Fig 2e and f (especially 2e). Anaphase onset is easy to see (and I presume they are using this rather than the harder- to-gauge timing of cytokinesis as the time of mitotic exit), but without a confident identification of NEBD or some other measure of the timing of mitotic onset, it is hard to be sure about mitotic duration. I can judge mitotic onset better from the movies, although it is chromatin condensation I am gauging, not NEBD. Perhaps if the authors measure the DNA staining fluorescence intensity as a function of time, they will be able to graphically show when chromatin condensation happens from a sudden increase in brightness. Or perhaps higher magnification microscopy would help. Or a different mitotic marker.

© 2022. Published by The Company of Biologists under the terms of the Creative Commons Attribution License (https://creativecommons.org/licenses/by/4.0/).

Response: It is true that chromosomal condensation is an important part of determining the time of NEBD but, in addition, the time at which nuclei transition from a smooth-outlined interphase shape to an irregularly-outlined collection of individual chromosomes is used to determine NEBD. In short, NEBD is defined as the frame prior to the time at which the outline of the stained DNA becomes irregular, provided that the cell also exhibits chromosome condensation as defined by visually increased DNA staining intensity and/or individualising chromosomes. Anaphase onset is defined as the first frame in which two chromosome masses can be seen moving towards the cell poles. These definitions are now made clearer in the manuscript (Figure 2D,E). We note that, even if this determination was inaccurate by a few movie frames, the error would be insignificant compared to the hours-long increase in the duration of mitosis observed here.

Additionally, as these live-cell imaging experiments have been carried out by us in a different facility (Newcastle University, UK), it is currently not possible for us to repeat the same at a higher magnification or with a different mitotic marker, and we are therefore hoping that the clarification of our analysis criteria and background, as mentioned above, would address the reviewer's concerns.

- The authors say that PCTAIRE1 levels peak in mitosis. But in Figs 5a and b, it is still increasing (or at least not yet decreasing) in the latest time points. And they say there is a "marked" increase in its phosphorylation. If there is any increase in phosphorylation beyond what you would expect just from a gradual increase in abundance, it is subtle. And it is not demonstrated that the multiple putative PCTAIRE1 bands represent different levels of phosphorylation. This could and should be checked by treating samples with lambda phosphatase ± vanadate.

Response: We have performed this experiment multiple times using both U2OS as well as HeLa cells to take into consideration the difference across different cell lines and also any potential artefacts arising out of running the gel or during the transfer to nitrocellulose membrane for Western blotting. We agree that in some of our experiments PCTAIRE1 protein levels are still increasing till the last time point of the experiment. However, as that usually coincided with the saturation of cyclin B1 and phospho-CDK substrate levels as well, we concluded that PCTAIRE1 amounts also peak around the same time. To better represent this, we have quantified the levels of PCTAIRE1, cyclin B1, and phospho-CDK substrates and presented them in a graph that has been included in the revised manuscript (Figure 5A,B).

Further, with regards to the phosphorylation levels of PCTAIRE1, we again agree with the reviewer that the increase in phosphorylation is just subtle and have therefore used more appropriate and toned down wording in the revised manuscript accordingly.

- Given the background fluorescence in Fig 2, I am not convinced that the signal in Fig 6 represents PCTAIRE1. Does knocking down PCTAIRE1 make the signal go away?

Response: As explained for the comment above, the fluorescence in Fig. 2 (Revised manuscript Supplementary Information, Fig. S2) is that of endogenous PCTAIRE1 and, therefore, does not invalidate the data shown here.

Same is the case with PLK1 (now Fig. S3). Further, we have shown that the signal goes away upon knockdown of PCTAIRE1 by esiRNA in Fig. 8.

- Can the PCTAIRE1-esiRNA knockdown phenotype be rescued by expressing PCTAIRE1 from a different species, or by using siRNA made from the 3'-UTR and rescuing with a construct that lacks the 3'-UTR?

Response: It would certainly be interesting to look at PCTAIRE1 knockdown phenotype in this perspective. However, unfortunately, due to the existing pandemic situation, it has been difficult for us to obtain a PCTAIRE1 construct of any other species or new siRNAs for PCTAIRE1.

© 2022. Published by The Company of Biologists under the terms of the Creative Commons Attribution License (https://creativecommons.org/licenses/by/4.0/).

If the authors can firm-up these main experimental findings, I could support publication of this paper, but as it is I am intrigued but not convinced.

Minor points:

#3. Line 46: "PCTAIRE1, also known as cyclin dependent kinase 16 (CDK16 or PCTK1) (Malumbres et al., 2009), belongs to the poorly-characterized family of cyclin dependent kinases (CDKs) which includes PCTAIRE 1, 2 and 3 (Manning et al., 2002)." Since some members of the CDK family are well-characterized, how about change "the poorly-characterized family" to "a poorly-characterized sub-family".

Response: We thank the reviewer for this suggestion and have accordingly edited the manuscript.

#4. Line 96: I think the authors are missing a word--maybe "using"--before the word "PolST".

Response: We have added the suggested word in the revised manuscript.

#5. Line 107: What was the rationale behind expressing myristoylated kinases rather than unaltered kinases for the screen? I appreciate that for some kinases, e.g. Akt, forcing the kinase to the membrane is sufficient to activate the kinase, but I would think that only a few of the 200 kinases would be activated this way. Was there some other reason? It would be helpful for the authors to explain this.

Response: This point was also raised by the other reviewer (#2), and we have already provided the explanation for this query.

#6. Line 124: GAK expression appeared to rescue viability in Fig 1c, but it was not considered a positive. From the supplement, I think the rescue shown in Fig 1c proved not to be reproducible. If so this should be stated clearly in the main paper.

Response: This is a valid point and we have accordingly edited the manuscript for more clarity with regards to GAK.

#7. Fig 2i, 3g. These panels are not "dot blots".

Response: We have revised the manuscript by using the correct term for the panels, "dot plots" instead of "dot blots" for figures 2H and 3F in the revised manuscript.

#8. Fig 4. Here the authors show that PCTAIRE1 overexpression renders cells less sensitive to taxol and nocodazole. Can the authors estimate how overexpressed, on average, PCTAIRE1 is relative to the endogenous protein?

Response: For most of our experiments, the relative percentage of expression of exogenous PCTAIRE1 with respect to endogenous protein was around 3-fold on average.

#9. Line 632: "were the cells were" should read "where the cells were".

Response: We have accordingly edited the revised manuscript.

© 2022. Published by The Company of Biologists under the terms of the Creative Commons Attribution License (https://creativecommons.org/licenses/by/4.0/).

Second decision letter

MS ID#: JOCES/2021/258831

MS TITLE: PCTAIRE1 promotes mitotic progression and resistance against antimitotic and apoptotic signals

AUTHORS: Shaida Andrabi, Syed Qaaifah Gillani, Irfana Reshi, Nusrat Nabi, Misbah Un Nisa, Zarka Sarwar, Sameer Ahmed Bhat, Thomas M Roberts, and Jonathan M.G. Higgins
ARTICLE TYPE: Research Article

We have now reached a decision on the above manuscript.

To see the reviewers' reports and a copy of this decision letter, please go to: https://submit-jcs.biologists.org and click on the 'Manuscripts with Decisions' queue in the Author Area. (Corresponding author only has access to reviews.)

As you will see, the reviewers gave favourable reports but raised some critical points that will require amendments to your manuscript. I hope that you will be able to carry these out because I would like to be able to accept your paper, depending on further comments from reviewers.

We are aware that you may be experiencing disruption to the normal running of your lab that makes experimental revisions challenging. If it would be helpful, we encourage you to contact us to discuss your revision in greater detail. Please send us a point-by-point response indicating where you are able to address concerns raised (either experimentally or by changes to the text) and where you will not be able to do so within the normal timeframe of a revision. We will then provide further guidance. Please also note that we are happy to extend revision timeframes as necessary.

Please ensure that you clearly highlight all changes made in the revised manuscript. Please avoid using 'Tracked changes' in Word files as these are lost in PDF conversion.

I should be grateful if you would also provide a point-by-point response detailing how you have dealt with the points raised by the reviewers in the 'Response to Reviewers' box. Please attend to all of the reviewers' comments. If you do not agree with any of their criticisms or suggestions please explain clearly why this is so.

Reviewer 1

Advance summary and potential significance to field

The authors

Comments for the author

Figure S5 (S4?): The authors claimed that the expression level of PCTAIRE1 is high in mitosis phase. The authors have not performed the cell cycle analysis by FACS.
Instead, they examined the mitotic cell ratio in U2OS cells through immunofluorescence analysis of pHH3 . Basically, PCTAIRE1 expression levels appear to increase as M-phase cells increase. Please add the levels of PCTAIRE1 at 16, 24 hours after thymidine block release (M phase - G1 phase). Did you observe the decrease of PCTAIRE1 after M phase?

Reviewer 3

Advance summary and potential significance to field

The authors show that PCTAIRE1 overexpression inhibits PolST-induced mitotic arrest, and it makes the case for PCTAIRE1 as a mitotic regulator.

© 2022. Published by The Company of Biologists under the terms of the Creative Commons Attribution License (https://creativecommons.org/licenses/by/4.0/).

Comments for the author

The revised ms adds some new data and makes some appropriate wording changes. I am still not convinced about the specificity of the PCTAIRE1 IF staining, though, and I think a couple of additional wording tweaks would be good.

1.The PCTAIRE1 blots in Fig 4A and D show essentially no signal in the control MDA-MB-468 and U2OS cells. However, by IF, the cells transfected with PolST without PCTAIRE1 have bright PCTAIRE1 signal (now in Figure S2). Possibly it is not quite as bright as in the PCTAIRE1-transfected cells, but I would need to see some careful quantification to be convinced of that. In their rebuttal, the authors say this IF signal is due to endogenous PCTAIRE1. But if the signal is similar ± PCTAIRE1 transfection by IF, wouldn't it be similar ± transfection by immunoblotting? The discordance between the IF and the immunoblotting results raises the possibility that some or much of the IF signal is not due to PCTAIRE1.

This concern is partially alleviated by the PCTAIRE1 knockdown experiment now shown in Fig 8A--qualitatively the signal looks brighter in the one control cell shown than it does in the PCTAIRE1-knockdown cells. It would be much more convincing if the authors showed a field of control cells vs. a field of knockdown cells, with the IF processed in parallel and the exposures set to be the same for both conditions.

And, if the field of knockdown cells does show the hoped-for ~70% decrease in PCTAIRE1 IF signal (as the western blot does), then the authors should explicitly discuss all these findings--e.g. something like this:
"The similar IF signals in the untransfected vs. PCTAIRE1-transfected raised the possibility that some or much of the IF signal was due to something other than PCTAIRE1. However, knocking down PCTAIRE1 with esiRNAs and/or shRNAs decreased both the IF signal and the immunoblotting signal to a similar degree. This supports the specificity of the immunostaining"

2.The authors nicely explain in the rebuttal how they came to use the myristoylated library, but they do not explain it in the paper. An explanation would help the reader to understand the experimental logic and should be added to the paper.

3.Like the authors, I do suspect that the 2-4 PCTAIRE1 bands seen in their western blots represent different phosphostates of the PCTAIRE1 protein, but since the authors have not done the lambda phosphatase experiment, they at least need to acknowledge explicitly that this is an assumption, not a demonstrated fact.

Second revision

Author response to reviewers' comments

Response to the queries of reviewers:

Reviewer 1 Advance Summary and Potential Significance to Field...

Reviewer 1 Comments for the Author...

Figure S5 (S4?): The authors claimed that the expression level of PCTAIRE1 is high in mitosis phase. The authors have not performed the cell cycle analysis by FACS. Instead, they examined the mitotic cell ratio in U2OS cells through immunofluorescence analysis of pHH3. Basically, PCTAIRE1 expression levels appear to increase as M-phase cells increase. Please add the levels of PCTAIRE1 at 16, 24 hours after thymidine block release (M phase - G1 phase). Did you observe the decrease of PCTAIRE1 after M phase?

Response: This is in fact a very valid question that we had already asked and addressed when the

© 2022. Published by The Company of Biologists under the terms of the Creative Commons Attribution License (https://creativecommons.org/licenses/by/4.0/).

manuscript was under revision. We wanted to know what happens to PCTAIRE1 levels during the course of a full cell cycle, and whether its levels would be reversed at the mitotic exit. Results indeed showed that at the mitotic exit, PCTAIRE1 levels were decreased, which validated our results. We have now provided the Western blots in Figure A (below) that shows this decrease in PCTAIRE1 levels when cells exit M phase, as is reflected by different mitotic markers.

Reviewer 3 Advance Summary and Potential Significance to Field...

The authors show that PCTAIRE1 overexpression inhibits PolST-induced mitotic arrest, and it makes the case for PCTAIRE1 as a mitotic regulator.

Reviewer 3 Comments for the Author...

The revised ms adds some new data and makes some appropriate wording changes. I am still not convinced about the specificity of the PCTAIRE1 IF staining, though, and I think a couple of additional wording tweaks would be good.

#1. The PCTAIRE1 blots in Fig 4A and D show essentially no signal in the control MDA-MB-468 and U2OS cells. However, by IF, the cells transfected with PolST without PCTAIRE1 have bright PCTAIRE1 signal (now in Figure S2). Possibly it is not quite as bright as in the PCTAIRE1- transfected cells, but I would need to see some careful quantification to be convinced of that. In their rebuttal, the authors say this IF signal is due to endogenous PCTAIRE1. But if the signal is similar ± PCTAIRE1 transfection by IF, wouldn't it be similar ± transfection by immunoblotting? The discordance between the IF and the immunoblotting results raises the possibility that some or much of the IF signal is not due to PCTAIRE1.

This concern is partially alleviated by the PCTAIRE1 knockdown experiment now shown in Fig 8A--qualitatively the signal looks brighter in the one control cell shown than it does in the PCTAIRE1-knockdown cells. It would be much more convincing if the authors showed a field of control cells vs. a field of knockdown cells, with the IF processed in parallel and the exposures set to be the same for both conditions.

And, if the field of knockdown cells does show the hoped-for ~70% decrease in PCTAIRE1 IF signal (as the western blot does), then the authors should explicitly discuss all these findings--e.g. something like this:
"The similar IF signals in the untransfected vs. PCTAIRE1-transfected raised the possibility that some or much of the IF signal was due to something other than PCTAIRE1. However, knocking down PCTAIRE1 with esiRNAs and/or shRNAs decreased both the IF signal and the immunoblotting signal to a similar degree. This supports the specificity of the immunostaining"

Response: We believe that this difference in PCTAIRE1 signal in the IF and Western Blotting results that are referred to here is mainly because these experiments were carried out at different time points and places. In fact, to maintain the low passage numbers of PCTAIRE1 cell lines, we made new stable cell lines of PCTAIRE1 from time to time, which can explain the difference in the ratios of PCTAIRE1 expression in the IF (which were carried out in the laboratory of Prof. Jonathan Higgins at Newcastle University, UK) *vs.* the particular Western blotting results referred to, the experiment for which was carried much later (which were carried out in the laboratory of Prof. Thomas Roberts at DFCI, USA). These experiments were carried out when Syed Qaaifah Gillani (first author) was on visiting fellowships in the two labs/institutes.

Additionally, the difference in the intensity of PCTAIRE1 in Fig. S2 (between PolST+dox (control) and PolST/PCTAIRE1+dox) is not that striking mainly because of the predominant rounded-up morphology of the cells in the control cells, which occurs due to mitotic progression as they are expressing PolST. We have always observed in immunofluorescence experiments (even in different experiments also) that the expression of all the proteins (even house keeping cytoskeletal proteins like actin, tubulin etc) appears much more intense in these rounded up mitotic cells than in the neighboring cells that are in interphase. This is also apparent in the PolST+dox panel of S2. The brightest ones in this panel are the rounded up mitotic cells, which are identified by the condensed nuclei as shown by DAPI staining. On the other hand, the non- mitotic cells in their vicinity (in the same filed of view/panel) have much lesser intensity for PCTAIRE1. In contrast, the intensity of

© 2022. Published by The Company of Biologists under the terms of the Creative Commons Attribution License (https://creativecommons.org/licenses/by/4.0/).

PCTAIRE1 expression is almost uniform in all the cells in the POLST/PCTAIRE1+dox. Though the intensity is slightly more prominent in the rounded up cells, but almost all the non-mitotic cells have certainly much higher PCTAIRE1 intensity than in the control (PolST+dox) cells. This difference can be much appreciated in the merged files of the two panels (Figure S2).

This difference is even more appreciable in the absence of the PolST expression (-dox) of the same experiment. For the sake of simplicity, this part was not shown in the manuscript.

As you can see, the difference between PCTAIRE1 expression in the control (PolST-dox) and the PCTAIRE1 overexpressing cells (PolST/PCTAIRE1-dox) is much more apparent where there are almost no mitotic cells, in the absence of PolST expression (-dox). This difference is again very striking in the merged images. Further, as suggested by the reviewer, to substantiate our data, we have also quantified the IF signal of PCTAIRE1 in its overexpression cell lines and compared it with that in the control cells (Fig. B graphical representation).

Additionally, we have also provided fields from PCTAIRE1 control cells and PCTAIRE1 knockdown cells from the same experiment (Fig. 8A) under similar exposure conditions, and included the corresponding quantification results of PCTAIRE1 (Figure C).

#2. The authors nicely explain in the rebuttal how they came to use the myristoylated library, but they do not explain it in the paper. An explanation would help the reader to understand the experimental logic and should be added to the paper.

Response: As suggested, we have now included the reason behind using the myristoylated library in the revised manuscript.

#3. Like the authors, I do suspect that the 2-4 PCTAIRE1 bands seen in their western blots represent different phosphostates of the PCTAIRE1 protein, but since the authors have not done the lambda phosphatase experiment, they at least need to acknowledge explicitly that this is an assumption, not a demonstrated fact.

Response: As suggested by the reviewer, we have edited the manuscript to clarify this point.

The supporting figures for these explanations for the reviewers are shown as below:

[NOTE: *We have removed unpublished data that had been provided for the referees in confidence*.]

© 2022. Published by The Company of Biologists under the terms of the Creative Commons Attribution License (https://creativecommons.org/licenses/by/4.0/).

Response to the Reviewer 3:

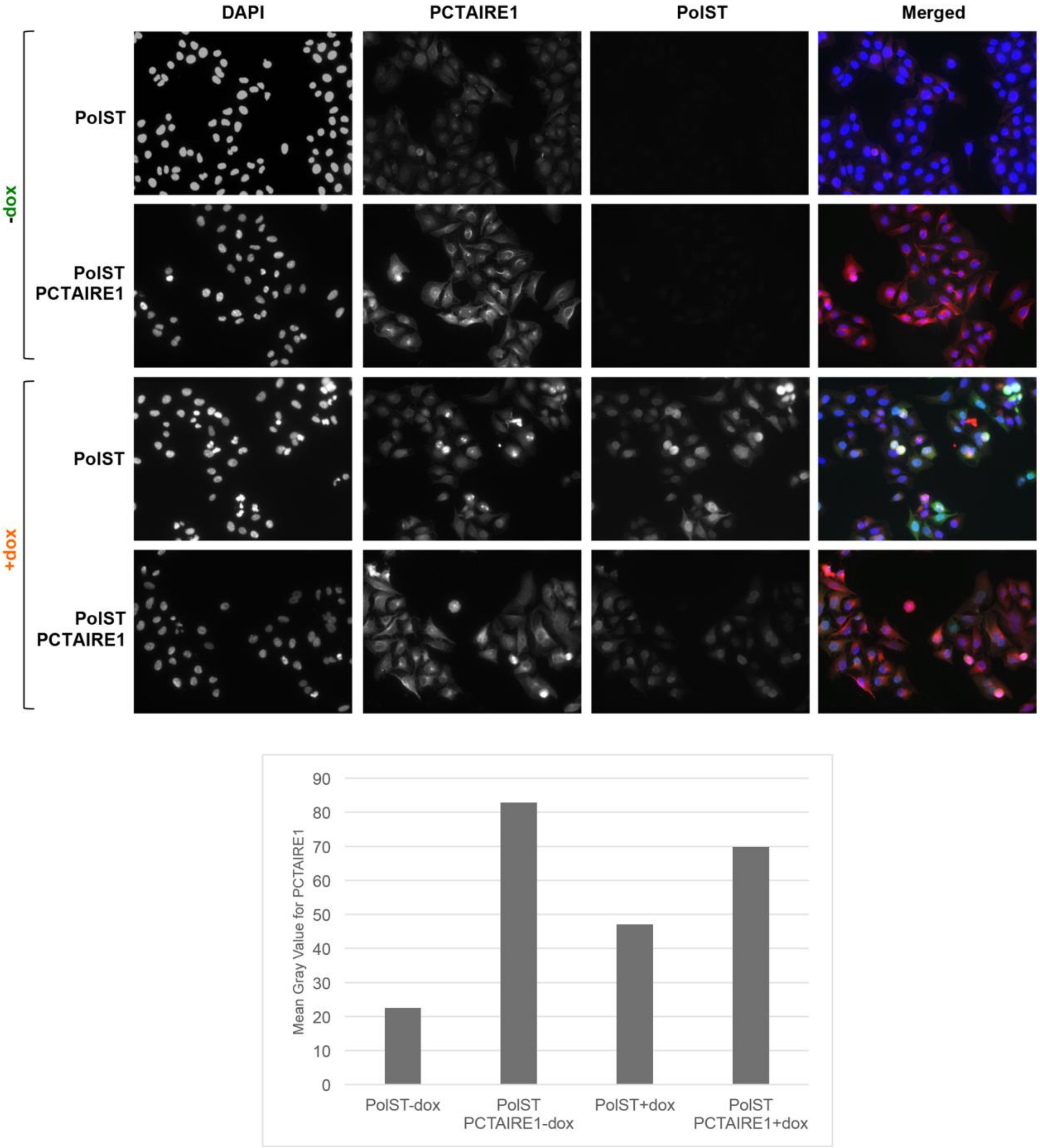

Figure B: Immunofluorescence analysis of PCTAIRE1 expression in control cells and PCTAIRE1 overexpressing cells in presence and absence of doxycycline for induction of PolST.

© 2022. Published by The Company of Biologists under the terms of the Creative Commons Attribution License (https://creativecommons.org/licenses/by/4.0/).

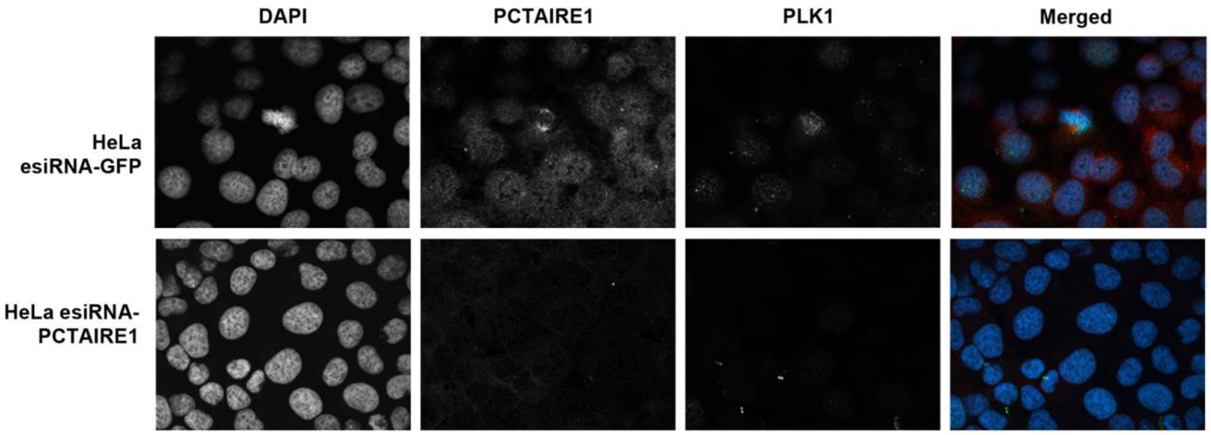

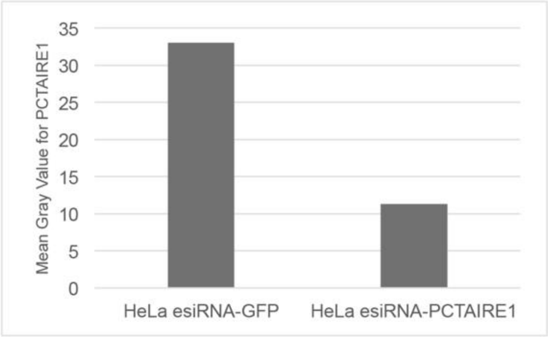

Figure C: Immunofluorescence analysis of PCTAIRE1 expression in control cells and PCTAIRE1 knockdown cells.

Third decision letter

MS ID#: JOCES/2021/258831

MS TITLE: PCTAIRE1 promotes mitotic progression and resistance against antimitotic and apoptotic signals

AUTHORS: Shaida Andrabi, Syed Qaaifah Gillani, Irfana Reshi, Nusrat Nabi, Misbah Un Nisa, Zarka Sarwar, Sameer Ahmed Bhat, Thomas M Roberts, and Jonathan M.G Higgins
ARTICLE TYPE: Research Article

I am happy to tell you that your manuscript has been accepted for publication in Journal of Cell Science, pending standard ethics checks.

© 2022. Published by The Company of Biologists under the terms of the Creative Commons Attribution License (https://creativecommons.org/licenses/by/4.0/).
